# Longitudinal relations between parenting stress and child internalizing and externalizing behaviors: Testing within-person changes, bidirectionality and mediating mechanisms

**DOI:** 10.3389/fnbeh.2022.942363

**Published:** 2022-12-16

**Authors:** Willeke Van Dijk, Marleen H. M. de Moor, Mirjam Oosterman, Anja C. Huizink, Karen Matvienko-Sikar

**Affiliations:** ^1^Department of Clinical, Neuro and Developmental Psychology, Amsterdam Public Health Research Institute, Vrije Universiteit Amsterdam, Amsterdam, Netherlands; ^2^Department of Psychology, Education and Child Studies, Erasmus School of Social and Behavioural Sciences, Erasmus University Rotterdam, Rotterdam, Netherlands; ^3^Department of Clinical Child and Family Studies, Amsterdam Public Health Research Institute, Vrije Universiteit Amsterdam, Amsterdam, Netherlands; ^4^School of Public Health, University College Cork, Cork, Ireland

**Keywords:** parenting behavior, child behavioral problems, bidirectional relations, random intercept cross-lagged panel model, parenting stress

## Abstract

**Introduction:**

Parenthood can be experienced as a pleasant but challenging period for parents, possibly accompanied by parenting stress. Early parenthood in particular is a vulnerable period as many parents experience biological and psychosocial changes related to new parenthood. Previous studies have shown that parenting stress is related to child behavior problems, but few studies have investigated the transactional relations across time between parenting stress and child internalizing and externalizing outcomes separately, examining within-person changes. The first aim of this study was to examine the transactional within-person associations of parenting stress and child internalizing and externalizing behavior problems across childhood from age 9 months to 9 years. As a second aim, we examined parenting as a possible underlying mechanism of the transactional associations by testing whether parental warmth and hostility mediate within-person associations of parenting stress and child behavior across time.

**Method:**

Data were analyzed from the Growing Up in Ireland longitudinal child cohort study including 7,208 caregiver-child dyads at wave 1 (child’s age 9 months), who were followed at child’s age three (wave 2), five (wave 3), and 9 years (wave 5). Primary caregiver’s and child’s age and gender, household income, occupational status, educational status, partner status, and cultural background were covariates assessed at all waves. Data were analyzed using a random intercept cross-lagged panel model (CLPM) in R-lavaan.

**Results:**

Bidirectional relations between parenting stress and child behavior were found for both internalizing and externalizing behavior from age 5 to 9, but not for earlier time points.

**Discussion:**

Our results did not indicate mediating effects of parental warmth or parental hostility in the associations between parenting stress and child behavior problems. Therefore, we conclude that parenting stress and child internalizing as well as parenting stress and child externalizing behaviors have transactional associations from child’s age 5 to 9 years. Future research examining transactional associations of parenting stress and child behaviors should investigate possible other mediations taking a within-person approach by utilizing the RI-CLPM.

## Introduction

Parenthood is often experienced as a pleasant but challenging period for parents. Raising children is an ongoing process as, when children grow older, their needs will develop and change (Sanders and Turner, 2018). Parents have to adapt to their children’s changing needs continuously. These needs entail actual skills that parents require for raising children and the need to be emotionally involved in their development. Besides these needs, parents often have to adapt to the changing social role in the family system. When parents do not have the resources available to adjust to these demands and changes, this may cause stress related to the parental role (Abidin, 1990; Deater-Deckard, 1998), which can be expressed in both psychological and physiological reactions (Jennings and Dietz, 2007). *Parenting stress* can be assessed using a variety of measures, with some scales consisting only of items asking about parent domains, such as the Parenting Stress Scale (PSS); (Berry and Jones, 1995) and others comprising child domains as well, including the Parenting Stress Index (PSI); (Abidin, 1995), and the Parenting Daily Hassles scale (PDH); (Crnic and Greenberg, 1990). Experiencing some levels of parenting stress is normal and almost unavoidable when parents adjust to changing demands and shifting roles (Leigh and Milgrom, 2008). However, when parenting stress does not diminish but persists, this can seriously impact parental mental health, parent-child relationships, and child development (Leigh and Milgrom, 2008; Vismara et al., 2016). Among parents with mental health disorders, such as depression and anxiety, parental stress may co-occur, which make the construct interrelated with other mental health constructs (Berry and Jones, 1995). Parenting stress may affect child development which can result in difficult child behaviors, including behavioral problems across various stages in childhood (Lessenberry and Rehfeldt, 2004). Parenting stress has been studied extensively in relation to *child behavioral problems*, with a predominant focus on parenting stress as a precursor of child behavioral outcomes,

suggesting that parenting stress may result in child maladaptive outcomes (Crnic et al., 2005; Tsotsi et al., 2019). In addition, possible dynamic transactional relations between parenting stress and child outcomes have been understudied. A theory that supports the possible transactional associations between parenting stress and child behaviors is the coercion theory of Patterson (Patterson and Oregon, 1982). This theory proposes that children’s difficult behavior is reinforced by negative parenting, for instance as a consequence of high levels of parenting stress, which in turn evokes more negative reactions and behaviors from the parent, thereby continuing in a cycle until one of the behaviors diminishes. It is highly important to investigate the transactional associations between parenting stress and *child internalizing* and *externalizing behaviors* during childhood as early behavioral problems can develop into more serious and persistent behavioral problems and detrimental health outcomes later in life (Broidy et al., 2003; Campbell, 2006; Lorber and Egeland, 2015). Both internalizing and externalizing problems are rather common among children, with the onset around the preschool years (Egger and Angold, 2006). Research has found that, when internalizing symptoms appear during early childhood, symptoms tend to increase until late childhood (Colder et al., 2002; Gazelle and Ladd, 2003; Sterba et al., 2007). Externalizing symptoms tend to peak from 2 to 5 years old, after which these behaviors decrease throughout childhood (Alink et al., 2006; Tremblay, 2010; Cole et al., 2012; Olson et al., 2017). Several studies have looked at the transactional effects of parenting stress and child behavior (Neece et al., 2012; Mackler et al., 2015; Stone et al., 2016; Cherry et al., 2019; Kochanova et al., 2021). Some of them have focused on either child behavioral problems in general, or externalizing behavior problems as a separate construct (Neece et al., 2012; Mackler et al., 2015; Cherry et al., 2019). Cherry et al. (2019) investigated bidirectionality between parenting stress and children’s behavior problems in 835 parent-child dyads at three time points from age 1 to 3. They found that higher parenting stress at child’s age 1 was associated with more behavior problems when the child was 2 years old, whereas more behavior problems at age 1 were associated with higher parenting stress at age 2 and 3. Neece et al. (2012) showed similar results, with bidirectionality between parenting stress and general child behaviors in an older age group, including 144 children from 3 to 9 years of age. A study conducted by Mackler et al. (2015) followed a group of 404 children and their parents from child’s age 4 until 10 years, with a total of four time points, and found bidirectional associations between parenting stress and externalizing child behavior across all time points. Other studies looked at parenting stress and externalizing and internalizing behavior as separate constructs (Stone et al., 2016; Kochanova et al., 2021). Stone et al. (2016) examined the development and bidirectionality in a sample of 1,582 children aged 4–9 years. They showed no associations from internalizing problem behaviors to parenting stress across the three time points, whereas they did find that parenting stress at the first time point was related to internalizing behaviors at the following time point. Different results were found for the associations between parenting stress and externalizing problems, with some bidirectional associations found for boys as opposed to none for girls. Another transactional study that investigated both parenting stress and child internalizing and externalizing behaviors separately included a sample of children aged between 2 and 5 years at the first time point, followed for 6 years, using three time points (Kochanova et al., 2021). They showed that parenting stress at the second time point, when children were aged 3–6, was positively associated with child internalizing problems between age 7 and 11 years, but not the other way around. For externalizing behaviors, no cross-lagged associations with parenting stress were found. The latter two transactional studies showed that parenting stress may have specific transactional relations with each dimension of child behavioral problems across childhood, which may be related to the different developmental trajectories and varying risk factors of each domain (Mathiesen et al., 2009; Williams et al., 2009; Bongers et al., 2011; Weeks et al., 2014). Even though quite some research has been performed on the transactional relations between parenting stress and child behavioral problems, results are not always consistent among the studies. Moreover, different results for the transactional relations between parenting stress and either internalizing and externalizing behaviors emphasize the importance to distinguish between these two broad behavioral domains. Further, the literature has investigated transactional associations between parenting stress and child behaviors using a between-person approach, which has methodological pitfalls. Therefore, a within-person approach is highly needed. In addition, little is known about the possible underlying mechanisms that may explain the associations of parenting stress and child behaviors over time. Several studies have proposed that *parenting behaviors* play an important role in child behavioral development (Pinquart, 2017; Neel et al., 2018). As parenting stress might challenge parenting in a way such that parents with high levels of parenting stress might practice more dysfunctional parenting behaviors (Crnic et al., 2005; Neel et al., 2018), parenting behaviors might mediate the association of parenting stress and child behavioral outcomes. As supported by Patterson’s coercion model, parenting stress, parental behaviors, and child behavioral outcomes can be reciprocally associated, with parenting stress leading to less optimal parenting practices, which in turn results in difficult child behaviors, but also the other way around (Patterson and Oregon, 1982). Examples of parenting behaviors that might affect child behavioral development include the dimensions *parental warmth and supportiveness* and *parental hostility and negativity* (Anthony et al., 2005). Parental warmth and supportiveness refer to affectionate, engaging, and responsive parenting (Zubrick et al., 2014), and low parental warmth has been associated with children’s internalizing and externalizing behavior problems, and higher levels of parenting stress (Baumrind, 1971; Stormshak et al., 2000; Park and Dotterer, 2018; Rothenberg et al., 2020; de Maat et al., 2021). When parents feel stressed, they may experience difficulties with their emotional regulation, and therefore may not be as emotionally available for their children. As a consequence, highly stressed parents may find it challenging to show affection, leading to lower parental warmth, and more hostile behaviors toward their child. As a result of less warmth and more hostility in parenting, children may develop internalizing, and externalizing symptoms. Also, difficult child behaviors may cause parents to show less parental warmth and more parental hostility, which consequently could result in stress experienced about their parenting behaviors. In a longitudinal study by Park and Dotterer (2018), associations between parenting stress and parenting were found in parents with children aged 3–5. This study demonstrated that parental stress was predictive of warm parenting. Neel et al. (2018) showed in their systematic review that parental warmth was negatively associated with behavioral outcomes in preterm children, as displayed by fewer internalizing and externalizing problems. Parental hostility, characterized by displays of over-controlling, negative or hostile parenting (Anthony et al., 2005), has also been related to the development of internalizing and externalizing behavior problems in children and parenting stress (Chang et al., 2003; Bayer et al., 2011; Edwards and Hans, 2015; Stover et al., 2016; Pinquart, 2017), but with associations the other way around. For instance, Chang et al. (2003) found that harsh parenting was associated with more aggressive behaviors in a sample of 3–6 year old children. More strict discipline in parenting practices have further been associated with increased levels of parenting stress (Pinquart, 2017). In the transactional model examined by Cherry et al. (2019), it was shown that observed parental supportiveness, as assessed using parent-child interaction scales as a composite of the subscales sensitivity, positive regard, and cognitive stimulation, mediates the relation between parenting stress and general child behavioral problems. Parenting stress at age 1 was related to lower levels of parental supportiveness at age 2, which was then associated with increased general behavioral problems when children were 3 years old (Cherry et al., 2019). The other way around, general child behavior problems at age 1 was also associated with lower parental supportiveness at age 2, which was then associated with parenting stress at age 3. In the study of Mackler et al. (2015), self-reported negative parental reactions toward children’s behaviors was investigated as a mediator between parenting stress and child externalizing behaviors. Negative parental reactions can be seen as a component of parental hostile behaviors, as hostility is associated with externalization of anger and negativity (Bridewell and Chang, 1997). Mackler et al. (2015), however, did not find that negative parental reactions mediated between parenting stress and child externalizing behavior over time. It has also been found by several studies that child behavior was related to maladaptive parenting practices over time (Pinquart, 2017; Neel et al., 2018). Thus, it may be that child behavior problems, both internalizing and externalizing, are associated with low parental warmth and higher hostility, which in turn is related to increased parenting stress at a later time point. As no studies have yet investigated the possible mediating role of parental warmth and hostility between child internalizing and externalizing behavior separately and parenting stress, more research is needed to further explore this. This study adds to the literature for several other reasons. Firstly, few studies have used a transactional approach to investigate how parenting stress and child behavioral problems are associated over time (Neece et al., 2012; Mackler et al., 2015; Stone et al., 2016; Cherry et al., 2019; Kochanova et al., 2021). The results of these transactional studies demonstrate reciprocal effects over time between parenting stress and child behavior suggesting both child and parent effects. However, these previous studies all investigated transactional associations of parenting stress and child behavior over time using a cross-lagged panel model (CLPM). The CLPM has been widely criticized (Pardini, 2008; Lansford et al., 2018) and it has been shown that the cross-lagged effects are affected by the differential stability of the variables examined. Moreover, the CLPM models the between-person associations across time which may not reflect the within-person associations that researchers are typically interested in. As such, the conclusions that previous studies made with regard to the transactional associations of parenting stress and child behaviors can be questioned. An extension of the traditional CLPM has been developed (Pardini, 2008), which includes a random intercept that enables discrimination between within-person and between-person differences (RI-CLPM). As such, the RI-CLPM model shows the lagged relations that can only refer to fluctuations within persons, so that we can examine how changes in parenting stress and child behavior problems within persons are related over time while controlling for stable between-person differences in parenting stress and child behavior. Secondly, the current study is innovative as it explores whether parental warmth and hostility mediate the bidirectional associations of parenting stress and child internalizing and externalizing behaviors during childhood from age 3 to 9 years. Thereby, we are able to investigate parenting behavior as a potential underlying mechanism by which parenting stress might affect child behavior, and vice versa. A mediation model testing parental warmth and hostility has not been investigated before in a longitudinal study examining within-person changes from infancy until middle childhood. We hypothesized within-person transactional associations between parenting stress and child externalizing and internalizing problems, with increasing parenting stress predicting increasing child internalizing and externalizing problems and increasing child internalizing and externalizing problems predicting increasing parenting stress over time. Also, we expected that parental warmth and hostility would mediate the associations between parenting stress and later child internalizing and externalizing behaviors, as well as between child internalizing and externalizing problems and later parenting stress. More specifically, we expected that within-person increases in parenting stress predict decreases in less parental warmth and increases in hostility, which subsequently predicts increases in internalizing and externalizing behavior problems at later time points, and that increasing internalizing and externalizing behavior problems predict decreases in parental warmth and hostility, which in turn predict elevated levels of parenting stress at the following time point. The aims of this study were to examine the transactional within-person associations of parenting stress and child internalizing and externalizing behavior problems across childhood from age 9 months (wave 1), to child’s age 3 years (wave 2), 5 years (wave 3), and 9 years (wave 5) and to investigate whether parental warmth and hostility mediate within-person associations of parenting stress and child behavior across time.

## Materials and methods

### Design

The study is a secondary data analysis of a longitudinal cohort study with five waves, of which four are included in the current study; wave 1 (child aged 9 months), wave 2 (3 years), wave 3 (5 years), and wave 5 (9 years). Wave 4 (7 years) was excluded as data on parenting and child behavior were not assessed at this wave. At each wave, the primary caregiver filled out questionnaires through a computer-assisted personal interview (CAPI) or, in case of more sensitive questions, a computer-assisted self-interview (CASI). At wave 1, only parenting stress was assessed. At wave 2, 3, and 5, parenting stress, child internalizing, and externalizing behavior, and parental warmth and hostility were assessed. Moreover, time-invariant covariates (TIC’s; primary caregiver’s gender and cultural background) were assessed at wave 1 and time-varying covariates (TVC’s; primary caregiver’s age, child’s age, household income, occupational status, educational status, and partner status) were assessed at all waves.

### Participants and procedures

Data used in the current study were derived from the Growing Up in Ireland National Longitudinal Study of Children (GUI),^1^ a nationally longitudinal representative cohort study of children living in the Republic of Ireland. The study was approved by a dedicated Research Ethics Committee established by the Department of Health and Children of Ireland. Families with children registered on the Child Benefit Register born between December 1st 2007 and June 30th 2008 could be included, with a total of 41,185 eligible children (Quail et al., 2011). Of this sample, a systematic selection was made to yield a total sample of 11,134 parent-child dyads. From the 11,134 children, 7,208 parent-child dyads (wave 1) were included; for the remaining sample (3,926 dyads) the primary caregiver was different across the waves. At all four waves, the sample consisted of 7,208 dyads (8 male parents). Data collection of the first wave started in September 2008 until April 2009. Families with children aged 9 months who gave signed consent were systematically selected from the Irish Child Benefit Register and were followed until their children were 9 years old with four follow-up waves. For the current study, we selected the families with a parent (either male or female) who was the primary caregiver across all waves. Participants with missing information on all the main outcomes at all waves (parenting stress, child’s internalizing and externalizing behavior, parenting warmth, and hostility) were excluded.

### Measures

#### Demographics

Information regarding socio-demographic factors (child’s age, term status, primary caregiver’s age (16–29, 30–30, 40 >), partnered (yes/no), household income (annual income in euros, presented in quintiles), occupational status (employed/not employed), and educational level (up to third level of education/third level of education or higher), and cultural background (white/non-white) were obtained from all participants.

#### Parenting stress

Parenting stress of the primary caregiver was measured with the 6-item Parental Stressors Scale of the PSS (5) at wave 1, 2, 3, and 5. Items were rated on a Likert scale (1 = strongly disagree to 5 = strongly agree). Examples of items are “Having children has meant having too few choices and too little control over my life” and “Caring for my child(ren) sometimes takes more time and energy than I have to give.” A total score of the subscale Parental Stressors was calculated by summing the scores on each item. Higher scores indicated higher overall parenting stress. The total PSS scale in the sample of this study has shown acceptable internal consistency (Cronbach’s α = 0.78).

#### Children’s emotional and behavioral problems

Children’s emotional and behavioral problems were assessed by the Strengths and Difficulties Questionnaire (SDQ); (Goodman, 1997), which was completed at waves 2, 3, and 5 by the primary caregiver. The SDQ consists of 25 items divided into the five subscales Emotional Symptoms, Conduct Problems, Hyperactivity, Peer Problems, and Prosocial Behavior. Examples of items are “Considerate of other people’s feelings,” “Helpful if someone is hurt, upset or feeling ill,” “Picked on or bullied by other children.” Items were answered by “not true” (0), “somewhat true” (1), and “certainly true” (2). Scores on each domain ranged between 0 and 10. For the current study, the scores on the subscale Emotional Symptoms and Peer Problems were combined by summarizing the scores to create an “Internalizing” subscale, and the Conduct Problems and Hyperactivity subscale were combined by summarizing the scores to create an “Externalizing” subscale (Goodman et al., 2010). Higher scores on the subscales indicate more difficulties. The subscales demonstrated acceptable reliability (Internalizing Cronbach’s α = 0.73, Externalizing Cronbach’s α = 0.78; Goodman, 1997).

#### Parenting behavior

Parenting behavior was measured using an instrument from the Longitudinal Study of Australian Children (LSAC); (Zubrick et al., 2014) at wave 2, 3, and 5, reported by the primary caregiver. Two parenting dimensions “Warmth” and “Hostility” were assessed. The dimension Warmth measured the degree of warmth in the relationship with the child, using 6 items (e.g., “How often do you express affection by hugging, kissing and holding this child?”). Items were answered on a 5-point Likert scale ranging from “almost never” (1) to “always/almost/always” (5). A mean score was created by dividing the summed score on each item score by 6, yielding a total score from 1 to 5 with higher scores indicating high levels of parenting warmth. The dimension Hostility assessed the levels of hostility used in the relationship with the child, using 5 items (e.g., “I have raised my voice with or shouted at this child”). Items were answered on a 11-point Likert scale ranging from “not at all” (0) to “all the time” (10). A mean score was calculated by adding up the scores on each item and dividing it by 5 so that the total score ranges from 0 to 10. Higher scores indicate higher levels of hostility. The warmth and hostility subscales of the current study show good to acceptable reliability (Cronbach’s α = 0.88 and α = 0.68, respectively).

### Statistical analyses

#### Background analyses

IBM SPSS Statistics 27 was used to check the distribution of the data and to calculate descriptive statistics for all waves. First, data were inspected for errors and outliers were checked by means of histograms. As the sample size is large and multiple tests were conducted, a more conservative alpha level of 0.01 was utilized. Second, descriptive analyses were conducted for all study variables at each wave (child’s age, term status, primary caregiver’s gender and age, partner status, household income, occupational status, educational status, cultural background, see Tables 1, 2). Independent samples *t*-tests were performed to examine differences in the main outcomes for dichotomous categorical variables (primary caregiver’s gender, child’s gender, cultural background, education, and occupation) and to examine whether drop-outs (defined as participants with missing information on all main outcomes on a particular wave) differ from the remaining sample in primary caregiver’s age, parenting stress levels, child internalizing and externalizing problems, parental warmth and hostility, at each wave, when compared to the previous wave. Chi-square tests of independence were performed to test if drop-outs and the remaining sample differ in child’s gender, partner status and cultural background. Covariates were added to the main analyses if significant associations between the covariates and the study variables were found at one of the waves. Also, separate repeated measures ANOVA were conducted for complete data on parenting stress, child internalizing behaviors, child externalizing behaviors, parental warmth, and hostility to explore changes over time. All repeated measures ANOVA were corrected with Greenhouse-Geisser as the assumption of sphericity was violated. In case of significant differences across waves, *post hoc* analyses with Bonferroni correction were performed.

**TABLE 1:** Demographics and background information of the primary caregiver and the child.

	*n* (%)/M (*SD*)
**Gender child**	
Male	3,620 (50.20)
Female	3,588 (49.80)
**Gender primary caregiver**	
Male	8 (0.10)
Female	7,200 (99.90)
**Child’s gestational age at birth**	7,189
	39.52 (*2.05*)
**Primary caregiver’s cultural background**	
White	6,855 (95.10)
Non-white	335 (4.65)
Missing	18 (0.25)

**TABLE 2:** Demographics and background information of the primary caregiver and the child across the 4 waves.

	Wave 1	Wave 2	Wave 3	Wave 5
**Age PC, *n* (%)**	**Range**	**Range**	**Range**	**Range**
	16–29 1,957 (27.2)	16–29 1,164 (16.1)	16–29 706 (9.8)	20–39 2,529 (35.1)
	30 > 5,251 (72.8)	30 > 6,044 (83.9)	30 > 6,502 (90.2)	40 > 4,676 (64.9)
**Household’s annual income in quintiles, *n* (%)**				
0–20%	1,155 (16.0)	1,163 (16.1)	1,158 (16.1)	1,002 (13.9)
20–40%	1,144 (15.9)	1,227 (17.0)	1,235 (17.1)	1,223 (17.0)
40–60%	1,342 (18.6)	1,376 (19.1)	1,387 (19.2)	1,276 (17.7)
60–80%	1,587 (22.0)	1,460 (20.3)	1,477 (20.5)	1,485 (20.6)
80–100%	1,485 (20.6)	1,636 (22.7)	1,645 (22.8)	1,560 (21.6)
**Occupational status PC, *n* (%)**				
Not employed	2,753 (38.2)	2,937 (40.7)	2,892 (40.1)	2,238 (31.0)
Employed	4,453 (61.8)	4,271 (59.3)	4,314 (59.9)	4,961 (68.8)
Missing	2 (< 0)	−	2 (< 0)	9 (0.1)
**Education level PC, *n* (%)**				
Up to third level	4,413 (61.2)	4,164 (57.8)	4,271 (59.3)	4,135 (57.6)
Third level or higher	2,791 (38.7)	3,032 (42.1)	2,935 (40.7)	3,050 (42.3)
Missing	4 (0.1)	12 (0.2)	2 (< 0)	23 (0.3)
**Partner PC in household, *n* (%)**				
Yes	6,555 (90.9)	6,451 (89.6)	6,440 (89.3)	6,429 (89.2)
No	653 (9.1)	747 (10.4)	768 (10.7)	779 (10.8)

PC, primary caregiver.

#### Main analyses

**FIGURE 1:**
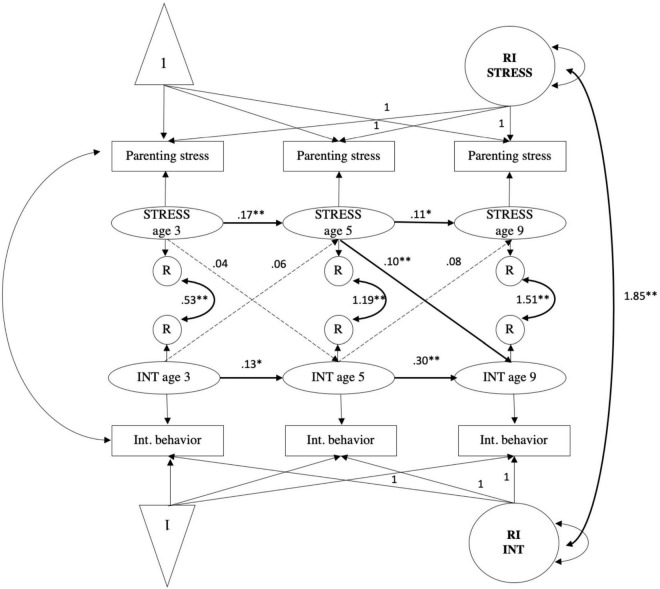
Random intercept cross-lagged panel model showing relations among parenting stress (STRESS) and child internalizing behaviors (INT) across three waves. Age 3, wave 2; age 5, wave 3; age 9, wave 5; R, residual variance; RI INT, random intercept child internalizing behavior; RI STRESS, random intercept parenting stress. Standardized estimates reported. Gray dashed paths indicated non-significant estimates. *p < 0.01, ^**^p < 0.001.

To investigate the within-person associations of parenting stress and child internalizing and externalizing behavior problems over time, random-intercept crossed lagged panel modeling (RI-CLPM) were used in R using the Rstudio software v.1.2.5019 and the Lavaan package (Rosseel, 2012). In the RI-CLPM (see for example Figure 1), random intercepts (between-person level) capture the time-invariant, trait-like stability of parenting stress, and child behavioral problems (either internalizing or externalizing behavior problems). The latent time-specific factors capture the within-person, state-like changes around its own expected score. Autoregressive and cross-lagged paths were estimated between the latent time-specific factors for parenting stress and child internalizing and externalizing behaviors. Moreover, the within-time correlations between parenting stress and child outcomes were estimated as well as the correlation between the random intercepts of parenting stress and child outcomes at the between-person level. Models were estimated with and without the covariates to examine whether results change after adjusting for the covariates. Primary caregiver’s cultural background and child’s gender were included in the model as TIC’s, and primary caregiver’s age, partner status, household income, occupational status, and educational level were included in the model as TVC’s (Mund et al., 2021). Model fit of the RI-CLPM models were compared with the classical CLPM models. Furthermore, model fit of the RI-CLPM models as a whole, as well as the statistical significance of the specific paths in the models were evaluated. The following fit indices to determine good model fit were used: the chi-square test (χ^2^), the Standardized Root Mean Square Residual (SRMR), where good fit is indicated by values of 0.08 or lower; the Tucker–Lewis Index (TLI; Tucker and Lewis, 1973) where good fit is indicated by values of 0.90 or higher; the Comparative Fit Index (CFI), where a good fit is indicated by values of 0.90 or higher; and the Root Mean Squared Error of Approximation Index (RMSEA), where acceptable fit is indicated by values of 0.08 or lower (Tucker and Lewis, 1973; Hu and Bentler, 1998; Mund et al., 2021). Consequently, the specific paths in the model with the best fit were examined by performing a *z*-test for the specific parameters. A full information maximum likelihood estimator (FIML) with a robust estimator was used. A maximum likelihood estimator (ML) with robust standard errors was used to handle missing values and adjust for any deviations from normality (Enders, 2001). To test the first research questions on the within-person bidirectional associations of parenting stress and child internalizing behaviors and child externalizing behaviors over time, we estimated the RI-CLPM in separate models. To examine the research question on the mediating effect of parenting warmth in the association of parenting stress and internalizing behavior, a trivariate RI-CLPM model with cross-lagged associations among parenting stress, parenting warmth, and child internalizing behavior was estimated. The same models were estimated for warmth and externalizing behavior, hostility and internalizing behavior, and hostility and externalizing behavior. In the mediation models, we tested whether the indirect paths, for example, from parenting stress to internalizing behavior *via* parental warmth, were significant. Additionally, parenting stress at wave 1 was included in the different models to test the paths from parenting stress at wave 1 to parenting stress, child internalizing behavior, externalizing behavior, parental warmth, and hostility at wave 2, and evaluate the significance of these paths.

## Results

Demographic and background information of the sample can be found in Tables 1, 2. The majority of primary caregivers were white and, except for one male primary caregiver, all primary caregivers were females. Descriptives of the main outcomes are reported in Table 3. All outcome variables correlated with each other across the waves (see Supplementary Table 6). Participants who did not complete wave 5 (*n* = 6) had lower child internalizing [*t* (5.18) = 9.97, *p* < 0.001], lower child externalizing [*t* (7,203) = 2.08, *p* = 0.010], and higher parental warmth scores [*t* (7,200) = −58.35, *p* < 0.001] at wave 3 compared to non-drop-outs. For the other waves, drop-outs (*n* = 2 in wave 2 and *n* = 1 in wave 3) did not differ from the remaining samples in scores on main outcomes, PC age, child’s gender, partner status or cultural background.

**TABLE 3:** Means and standard deviations of parental stress scores, child internalizing, child externalizing, parental warmth, and parental hostility of wave 1, 2, 3, and 5.

	Wave 1	Wave 2	Wave 3	Wave 5	*F*-test
Parenting stress, *n*	7,172	7,121	7,117	7,107	670.81**
M (SD)	14.53 (4.17)	12.23 (4.09)	11.67 (4.02)	13.41 (4.39)	
Child Internalizing, *n*	−	7,205	7,205	7,201	172.47**
M (SD)		2.45 (2.16)	2.45 (2.39)	2.96 (2.89)	
Child Externalizing, *n*	−	7,203	7,205	7,202	336.65**
M (SD)		5.14 (3.28)	4.65 (3.32)	4.15 (3.39)	
Parental warmth, *n*	−	7,206	7,207	7,119	732.03**
M (SD)		4.74 (0.37)	4.73 (0.39)	4.53 (0.58)	
Parental hostility, *n*	−	7,203	7,202	7,110	712.51**
M (SD)		1.79 (0.48)	1.80 (0.49)	2.02 (0.61)	

**Significance level of < 0.001. M (SD), mean (standard deviation).

### Background analyses

#### Associations between covariates and main outcomes

At each wave, at least for one of the main outcomes, including parenting stress, child internalizing behavior, child externalizing behavior, parental warmth, and parental hostility, significant associations with covariates were found (see Supplementary Tables 1–5). Therefore, we corrected for all covariates at all waves in our analyses.

#### Changes in parenting stress, internalizing, externalizing, parental warmth, and hostility from age 9 months, 3, 5 to 9 years

Repeated Measures ANOVA showed significant changes in parenting stress across waves [*F* (1, 2.80) = 1140.84, *p* < 0.001], with decreases in parenting stress from wave 1 (*M* = 14.53, *SD* = 4.17) to wave 2 (*M* = 12.23, *SD* = 4.10); to wave 3 (*M* = 11.67, *SD* = 4.02), and an increase from wave 3 to wave 5 (*M* = 13.41, *SD* = 4.39). For internalizing behaviors, a significant change was also found [*F* (1, 1.90) = 172.47, *p* < 0.001], with an increase in internalizing behaviors from wave 3 (*M* = 2.45, *SD* = 2.39) to wave 5 (*M* = 2.96, *SD* = 2.89). Also, for externalizing behaviors significant changes were found [*F* (1, 1.93) = 336.65, *p* < 0.001], with a decrease from wave 2 (*M* = 5.14, *SD* = 3.28) to wave 3 (*M* = 4.65, *SD* = 3.32); to wave 5 (*M* = 4.15, *SD* = 3.39). For parental warmth, significant changes were found [*F* (1, 1.75) = 732.03, *p* < 0.001], with a decrease from wave 2 (*M* = 4.74, *SD* = 0.37) to wave 3 (*M* = 4.73, *SD* = 0.39); to wave 5 (*M* = 4.53, *SD* = 0.58). For parental hostility, we also found significant changes [*F* (1, 1.87) = 712.25, *p* < 0.001], with increases from wave 2 (*M* = 1.79, *SD* = 0.48) to wave 3 (*M* = 1.80, *SD* = 0.49); to wave 5 (*M* = 2.02, *SD* = 0.61).

### Main analyses

#### Parenting stress and internalizing behavior

The CLPM fit measures were; χ^2^ (4) = 573.54, *p* < 0.001; CFI = 0.946, RMSEA = 0.141, SRMR = 0.041, TLI = 0.799. The fit of the RI-CLPM model was significantly better when compared to the CLPM (see Table 4); χ^2^ (1) = 37.50, *p* < 0.001; CFI = 0.997, RMSEA = 0.071, SRMR = 0.014, TLI = 0.949; ΔS-B χ^2^ (3) = 536.05, *p* < 0.001. The results of the RI-CLPM testing the within-person bidirectional associations between parenting stress and internalizing behavior are displayed in Figure 1. Significant auto-regressive paths were found for parenting stress from age 3 to 5 (B = 0.17, SE = 0.03, *p* < 0.001) and from age 5 to 9 (*B* = 0.11, *SE* = 0.03, *p* < 0.001). Also, for internalizing behavior significant auto-regressive paths were observed from age 3 to 5 (*B* = 0.13, *SE* = 0.04, *p* < 0.001) and from age 5 to 9 (*B* = 0.30, *SE* = 0.02, *p* < 0.001). A significant cross-lagged effect was found from parenting stress at age 5 to internalizing behavior at age 9 (*B* = 0.10, *SE* = 0.03, *p* < 0.001). After correction for covariates, only the significance of the path between internalizing behavior at age 5 and parenting stress at age 9 changed (*B* = 0.10, *SE* = 0.04, *p* < 0.01) (see Supplementary Figure 3). After including parenting stress at wave 1 in the model of parenting stress and child internalizing behavior (see Supplementary Figure 1), the path between parenting stress at age 3 to child internalizing behavior at age 5 was significant (*B* = 0.05, *SE* = 0.01, *p* < 0.001). After correction for covariates, the significance of none of the paths changed (see Supplementary Figure 9). Thus, within-person changes in parenting stress and internalizing behaviors were not bidirectionally associated from age 3 to 5, whereas they were from age 5 to 9 years.

**TABLE 4:** Model fit for all random-intercept cross lagged models with and without covariates included.

RI-CLPM	CFI	RMSEA	SRMR	TLI	χ^2^ *P*-value
Parenting stress-internalizing	0.997	0.071	0.014	0.949	< 0.001
Parenting stress-internalizing-Cov	0.898	0.072	0.130	0.898	< 0.001
Parenting stress-warmth-internalizing	0.996	0.054	0.013	0.947	< 0.001
Parenting stress-warmth-internalizing-Cov	0.902	0.067	0.117	0.859	< 0.001
Parenting stress-hostility-internalizing	0.998	0.041	0.011	0.974	< 0.001
Parenting stress-hostility-internalizing-Cov	0.905	0.067	0.118	0.863	< 0.001
Parenting stress-externalizing	1.000	0.000	0.001	1.001	0.76
Parenting stress-externalizing-Cov	0.928	0.076	0.113	0.870	< 0.001
Parenting stress-warmth-externalizing	0.999	0.022	0.007	0.993	< 0.01
Parenting stress-warmth-externalizing-Cov	0.905	0.067	0.118	0.864	< 0.001
Parenting stress-hostility-externalizing	0.998	0.042	0.010	0.979	< 0.001
Parenting stress-hostility-externalizing-Cov	0.909	0.067	0.118	0.870	< 0.001

RI-CLPM, random-intercept cross lagged panel model; CFI, comparative fit index; RMSEA, root mean squared error of approximation index; SRMR, standardized root mean square residual; TLI, Tucker Lewis index.

#### Parenting stress, internalizing behavior, and parental warmth

**FIGURE 2:**
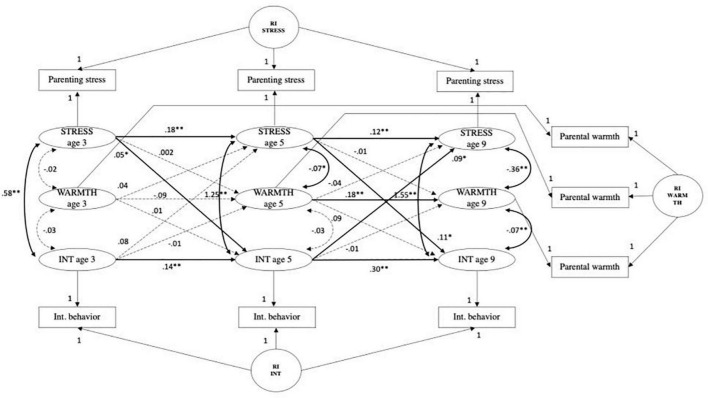
Random intercepts cross-lagged panel model showing relations among parenting stress (STRESS), parental warmth (WARMTH) and child internalizing behaviors (INT) across three waves. Age 3, wave 2; age 5, wave 3; age 9, wave 5; R, residual variance; RI INT, random intercept child internalizing behavior; RI STRESS, random intercept parenting stress; RI WARMTH, random intercept parental warmth. Standardized estimates reported. Gray dashed paths indicated non-significant estimates. *p < 0.01, ^**^p < 0.001.

The CLPM fit measures were; χ^2^ (9) = 824.89, *p* < 0.001; CFI = 0.942, RMSEA = 0.112, SRMR = 0.036, TLI = 0.770. The fit of the RI-CLPM model was significantly better compared to the CLPM (see Table 4); χ^2^ (3) = 66.17, *p* < 0.001; CFI = 0.996, RMSEA = 0.054, SRMR = 0.013, TLI = 0.947; ΔS-B χ^2^ (6) = 758.72, *p* < 0.001. Overall, the model showed that the associations between parenting stress and child internalizing behaviors were not mediated by parental warmth (see Figure 2). The paths from internalizing behavior at age 3 to parental warmth age 5, and from parental warmth age 5 to parenting stress at age 9, and vice versa, were not significant. Furthermore, both indirect effects were not significant. After including parental warmth in the model, the path from parenting stress at age 3 to age 5 was significant (*B* = 0.12, *SE* = 0.03, *p* < 0.001). Besides the significant autoregressive paths for parenting stress and internalizing behavior, a significant positive autoregressive path from parental warmth at age 5 to parental warmth at age 9 was observed (*B* = 0.18, *SE* = 0.03, *p* < 0.001). After correction for covariates, the path between parenting stress at age 5 to parental warmth at age 9 was significant (*B* = −0.01, *SE* = 0.003, *p* < 0.01) (see Supplementary Figure 4). Thus, parental warmth did not mediate between parenting stress and child internalizing behavior.

#### Parenting stress, internalizing behavior, and parental hostility

**FIGURE 3:**
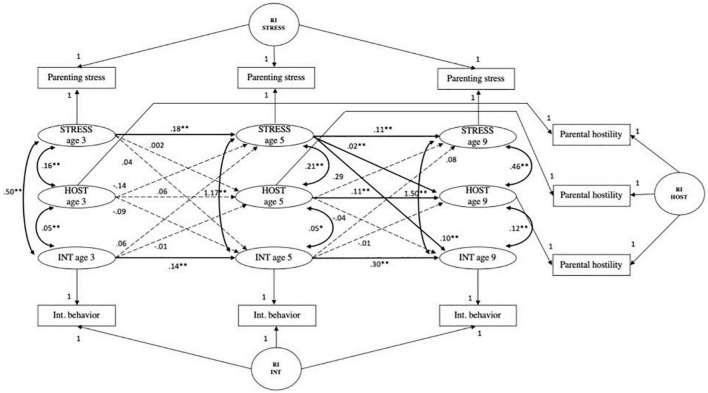
Random intercepts cross-lagged panel model showing relations among parenting stress (STRESS), parental hostility (HOST) and child internalizing behaviors (INT) across three waves. Age 3, wave 2; age 5, wave 3; age 9, wave 5; R, residual variance; RI INT, random intercept child internalizing behavior; RI STRESS, random intercept parenting stress; RI HOST, random intercept parental hostility. Standardized estimates reported. Gray dashed paths indicated non-significant estimates. *p < 0.01, ^**^p < 0.001.

The CLPM fit measures were; χ^2^ (9) = 812.48, *p* < 0.001; CFI = 0.952, RMSEA = 0.111, SRMR = 0.037, TLI = 0.810. The fit of the RI-CLPM model was significantly better when compared to the CLPM (see Table 4); χ^2^ (3) = 39.36, *p* < 0.001; CFI = 0.998, RMSEA = 0.041, SRMR = 0.011, TLI = 0.974; ΔS-B χ^2^ (6) = 773.12, *p* < 0.001. A significant positive autoregressive path from parental hostility at age 5 to parental hostility at age 9 was observed (*B* = 0.11, *SE* = 0.03, *p* < 0.001). Overall, the model showed that the associations between parenting stress and child internalizing behaviors were not mediated by parental hostility (see Figure 3). The paths from internalizing behavior age 3 to parental hostility age 5 to parenting stress at age 9 or vice versa, were not significant. Furthermore, both indirect effects were not significant. The path from parenting stress at age 5 to parental hostility at age 9, was found to be significant (*B* = 0.02, *SE* = 0.003, *p* < *0.001*). Significance of none of the paths in the model of parenting stress, internalizing behavior and parental warmth changed after correcting for covariates (see Supplementary Figure 5). Thus, parental hostility did not mediate between parenting stress and child internalizing behavior.

#### Parenting stress and externalizing behavior

**FIGURE 4:**
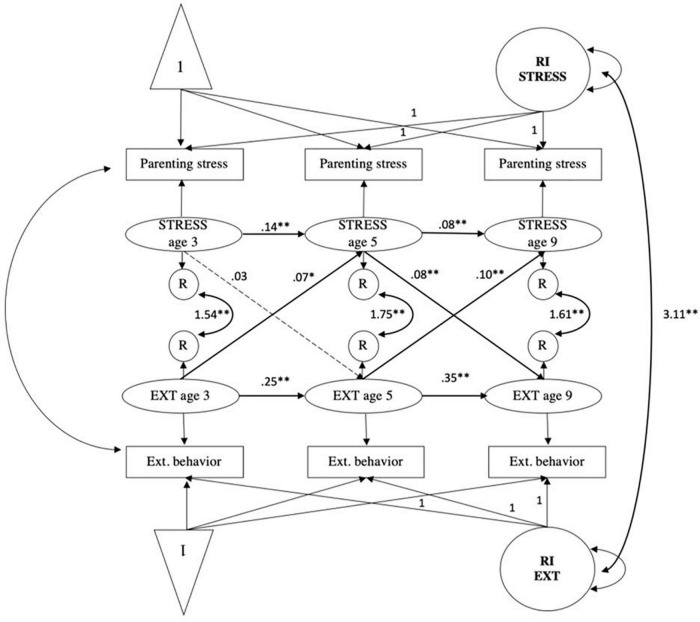
Random intercepts cross-lagged panel model showing relations among parenting stress (STRESS) and child externalizing behaviors (EXT) across three waves. Age 3, wave 2; age 5, wave 3; age 9, wave 5; R, residual variance; RI EXT, random intercept child externalizing behavior; RI STRESS, random intercept parenting stress. Standardized estimates reported. Gray dashed paths indicated non-significant estimates. *p < 0.01, ^**^p < 0.001.

The CLPM fit measures were; χ^2^ (4) = 588.33, *p* < 0.001; CFI = 0.957, RMSEA = 0.142, SRMR = 0.039, TLI = 0.838. The fit of the RI-CLPM model was significantly better when compared to the CLPM (see Table 4); χ^2^ (1) = 0.09, *p* = 0.76; CFI = 1.000, RMSEA = 0.000, SRMR = 0.000, TLI = 1.001; ΔS-B χ^2^ (3) = 588.24, *p* < 0.001. Significant positive auto-regressive paths were found for parenting stress from age 3 to 5 (*B* = 0.14, *SE* = 0.03 *p* < 0.001) and from age 5 to 9 (*B* = 0.08, *SE* = 0.03, *p* < 0.001). For externalizing behavior, significant positive auto-regressive paths were also observed from age 3 to 5 (*B* = 0.25, *SE* = 0.03, *p* < 0.001) and from age 5 to 9 (*B* = 0.35, *SE* = 0.02, *p* < 0.001). Overall, the RI-CLPM shows bidirectional associations between parenting stress and externalizing behavior from age 5 to 9 (see Figure 4). Cross-lagged effects were observed from age 5 parenting stress to age 9 externalizing behavior (*B* = 0.08, *SE* = 0.02, *p* < 0.001). Moreover, cross-lagged effects were observed from age 3 externalizing behavior to age 5 parenting stress (*B* = 0.07, *SE* = 0.03, *p* < 0.01), and from age 5 externalizing behavior to age 9 parenting stress (*B* = 0.10, *SE* = 0.03, *p* < 0.001). After correction for covariates, the autoregressive path from parenting stress at age 5 to parenting stress at age 9 was non-significant (*B* = 0.07, *SE* = 0.03, *p* = 0.02) (see Supplementary Figure 6). After including parenting stress at wave 1 in the model of parenting stress and child externalizing behavior (see Supplementary Figure 2), the path between parenting stress at age 3 to child externalizing behavior at age 5 was significant (*B* = 0.08, *SE* = 0.02, *p* < 0.001). After correction for covariates, the significance of none of the paths changed (see Supplementary Figure 10). Thus, within-person changes in parenting stress and externalizing behaviors were not bidirectionally associated from age 3 to 5, whereas it was from age 5 to 9 years.

#### Parenting stress, externalizing behavior, and parental warmth

**FIGURE 5:**
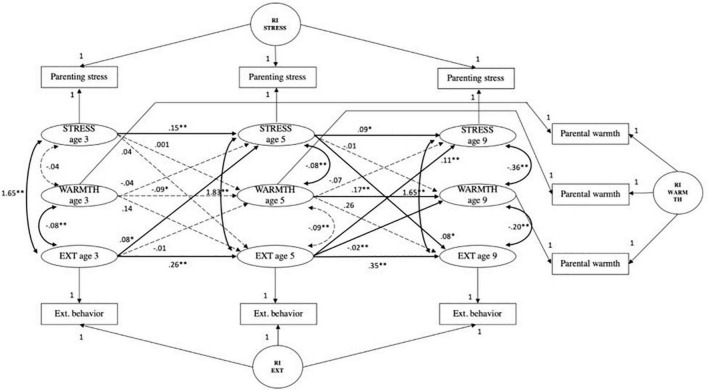
Random intercepts cross-lagged panel model showing relations among parenting stress (STRESS), parental warmth (WARMTH) and child externalizing behaviors (EXT) across three waves. Age 3, wave 2; age 5, wave 3; age 9, wave 5; R, residual variance; RI EXT, random intercept child externalizing behavior; RI STRESS, random intercept parenting stress; RI WARMTH, random intercept parental warmth. Standardized estimates reported. Gray dashed paths indicated non-significant estimates. *p < 0.01, ^**^p < 0.001.

The CLPM fit measures were; χ^2^ (9) = 832.02, *p* < 0.001; CFI = 0.952, RMSEA = 0.113, SRMR = 0.035, TLI = 0.809. The fit of the RI-CLPM model was significantly better when compared to the CLPM (see Table 4); χ^2^ (3) = 13.38, *p* ≤ 0.001; CFI = 0.999, RMSEA = 0.022, SRMR = 0.007, TLI = 0.993; ΔS-B χ^2^ (6) = 818.64, *p* < 0.001. A significant positive autoregressive path from parental warmth at age 5 to parental warmth at age 9 was observed (*B* = 0.18, *SE* = 0.03, *p* < 0.001). Overall, the model indicates no mediation effect of parental warmth in the association between parenting stress and externalizing behavior (see Figure 5). The paths from externalizing behavior age 3 to parental warmth age 5 and from parental warmth age 5 to parenting stress at age 9, and vice versa, were not significant. Furthermore, both indirect effects were not significant. The path from externalizing behavior at age 5 to parental warmth at age 9 was found to be significant (*B* = −0.02, *SE* = 0.003, *p* < 0.001). None of the paths in the model of parenting stress, externalizing behavior and parental warmth changed after correcting for covariates (see Supplementary Figure 7).

#### Parenting stress, externalizing behavior, and parental hostility

**FIGURE 6:**
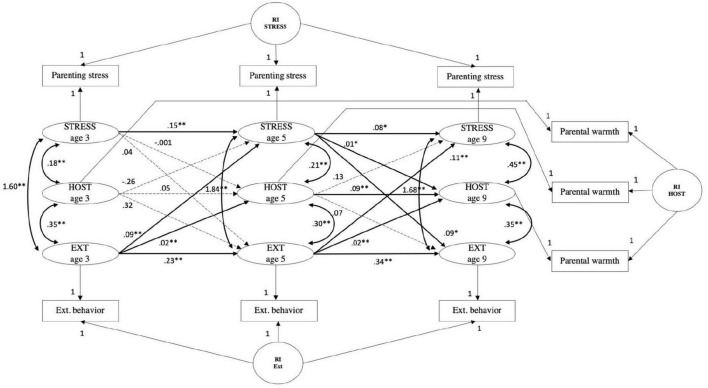
Random intercepts cross-lagged panel model showing relations among parenting stress (STRESS), parental hostility (HOST) and child externalizing behaviors (EXT) across three waves. Age 3, wave 2; age 5, wave 3; age 9, wave 5; R, residual variance; RI EXT, random intercept child externalizing behavior; RI STRESS, random intercept parenting stress; RI HOST, random intercept parental hostility. Standardized estimates reported. Gray dashed paths indicated non-significant estimates. *p < 0.01, ^**^ *p* < 0.001.

The CLPM fit measures were; χ^2^ (9) = 845.47, *p* < 0.001; CFI = 0.961, RMSEA = 0.114, SRMR = 0.036, TLI = 0.846. The fit of the RI-CLPM model was significantly better when compared to the CLPM (see Table 4); χ^2^ (3) = 40.97, *p* ≤ 0.001; CFI = 0.998, RMSEA = 0.042, SRMR = 0.010, TLI = 0.979; ΔS-B χ^2^ (6) = 884.51, *p* < 0.001. A significant positive autoregressive path from parental hostility at age 5 to parental hostility at age 9 was observed (*B* = 0.09, *SE* = 0.03, *p* < 0.001). Overall, the model indicates no mediation effect of parental hostility on the association between parenting stress and externalizing behavior (see Figure 6). In the pathway from externalizing behavior *via* parental hostility to parenting stress, one of the paths, from externalizing behavior age 3 to parental hostility age 5, was significant (*B* = 0.02, *SE* = 0.004, *p* < 0.001), whereas the path from parental hostility age 5 to parenting stress at age 9 was not significant. Within-person increases in parental hostility at age 5 were predicted by within-person increases in externalizing behavior at age 3, whereas within-person deviations in parenting stress at age 9 were not predicted by within-person deviations in parental hostility at age 5. Furthermore, both indirect effects were not significant. None of the paths in the model of parenting stress, externalizing behavior and parental hostility changed after correcting for covariates (see Supplementary Figure 8).

## Discussion

The current study aimed to examine the transactional associations between within-person changes in *parenting stress* and *child internalizing* and *externalizing behavior* across childhood from age 9 months to 9 years using the novel RI-CLPM. Our second aim was to test whether parenting behaviors, specifically *parental warmth* and *hostility*, mediated the within-person cross-time associations between parenting stress and child behavioral outcomes. Our results demonstrate that parenting stress at age 5 predicted child internalizing behaviors at age 9. Child externalizing behaviors at age 3 predicted parenting stress at age 5 and transactional associations between parenting stress and externalizing behaviors from age 5 and 9 were found. Neither parental warmth nor parental hostility mediated any of these associations. However, within-person cross-time associations were found between parenting stress at age 5 and parental hostility at age 9 in the model of internalizing behaviors, as well as between externalizing behavior and parental hostility from age 3 to age 9. The results of the current study partly support our first hypotheses that parenting stress and child internalizing and externalizing behaviors show bidirectional associations over time. We found transactional associations between parenting stress and both internalizing and externalizing behaviors from age 5 to 9 after correction for covariates. These results suggest that increases in parenting stress at age 5 predicts both increases in child internalizing as well as child externalizing behaviors at age 9. Also, converse associations were demonstrated such that increases in child internalizing behaviors, as well as in externalizing behaviors, at age 5 predicted increases in parenting stress at age 9. This finding is particularly interesting, since the predictive effect of child behavior on parenting stress is much less extensively studied. Possibly, when a parent notices increases in more difficult child behaviors, this may result in more stress experienced by the parent, for instance since the parent may feel overwhelmed by the increase in demands in parenting needed to deal with these behaviors. No transactional associations were found between parenting stress and child internalizing from child’s age 3–5 years old. These findings relate to those of Stone et al. (2016), who did not find transactional associations between parenting stress and internalizing behaviors between the ages 4–9. Their results suggest only parent effects, with parenting stress at age 4–7 years predicting internalizing behaviors 1 year later. This parent effect is consistent with our results that parenting stress at age 5 predicted internalizing behaviors at age 9. Also, Kochanova et al. (2021) found that parenting stress when children were aged between 3 and 6 was positively associated with child internalizing problems between ages 7 and 11, suggesting parent effects, and not the other way around. The most striking inconsistency between our findings and those of Stone et al. (2016) and Kochanova et al. (2021) is that, besides parenting effects, we also found child effects from child internalizing behaviors to parenting stress from age 5 to 9 after correction for covariates. A first explanation for the discrepancy in findings in the models of parenting stress and child behaviors is that these previous studies used the classical CLPM instead of the more advanced RI-CLPM that we used in our study. To illustrate the differences between the two methods, we tested the models using both methods and compared model fit. These results clearly indicated worse model fit for the CLPM models when compared to the RI-CLPM. Secondly, differences in covariates assessed and corrected for by the different studies may explain the discrepancies in findings. Changes in covariates over time, such as in the primary caregiver’s occupational status or partner status, may account for the association between internalizing behaviors to later parenting stress. The partner status of the primary caregiver at child’s age 5 years, for example being a single parent, may have been associated with increased child internalizing behaviors at age 5. Suppose that this parent is in a relationship 4 years later, when the child is 9 years old, and parenting stress may have been decreased. It could be that parenting stress has been reduced as a consequence of this change in partner status as now, the parent receives support from the partner. Thus, parenting stress would be associated with partner status. If we would not have controlled for partner status, we would not have found an association between internalizing at age 5 and later parenting stress, since internalizing behaviors were high at age 5 and parenting stress was low at age 9. Stone et al. (2016) solely corrected for maternal health and age, whereas Kochanova et al. (2021) controlled for, among others, number of people in the household, and child age and gender, but not for occupational status, partner status, educational status, and household income like we did in our study. Transactional associations were also demonstrated between parenting stress and externalizing behavior from ages 5 to 9, whereas only a positive association was found from externalizing behaviors at age 3 to parenting stress at age 5. These results are predominantly in agreement to those of Mackler et al. (2015) who found transactional associations between parenting stress and externalizing behavior across a period from child’s age 4–10 years. Kochanova et al. (2021) did not find bidirectionality between parenting stress and externalizing behaviors at all, whereas Stone et al. (2016) found different results for the associations between parenting stress and externalizing problems between boys and girls, with some transactional associations found for boys as opposed to none for girls. Even though we did not examine the models for boys and girls separately, we did correct for the child’s gender in our analyses and thereby accounted for possible differences in associations based on the child’s gender. As for the studies on the association between parenting stress and internalizing behaviors, the inconsistencies in findings between the studies may be due to the difference in analytical methods used, as our RI-CLPM specifically looked at within-person associations, whereas the CLPM as used by the other studies did not distinguish between within-person and between-person effects (Pardini, 2008). Interestingly, bidirectionality between parenting stress and child internalizing as well as externalizing behaviors was found from age 5 to 9, and not from age 3 to 5. As opposed to externalizing behaviors, internalizing behaviors could easily go unnoticed by parents, and maybe also because at very young ages children’s verbal skills are less well-developed yet, which makes them less competent in expressing their internal feelings (Tandon et al., 2009). If parents do not notice child’s internalizing behaviors, it is not predictive of later parenting stress during early childhood. Externalizing behaviors at age 3 predicted parenting stress at age 5, which was not found for internalizing behaviors, which may imply that acting-out behaviors are in general easier to notice and possibly are more disruptive for the parents thereby causing higher parenting stress as opposed to more inward-directed behaviors. This idea is also supported by studies examining correspondence between different informants, with often larger correspondence found in reports of children’s externalizing problems when compared to internalizing problems (Van der Ende et al., 2012; De Los Reyes et al., 2015). Disagreement in behavioral reports may be an indication of the difficulty of noticing specific behaviors. A study of Costa et al. (2006) investigated the specificity of parenting stressors and disentangled the associations between various parenting stressors and internalizing and externalizing behaviors, with parenting stress measured using the Parenting Stress Index-short form (PSI-SF; Abidin, 1990). This scale captures distress parents experience as a function of personal factors that are associated with parenting (parental distress), children’s temperamental and behavioral characteristics (difficult child), parental perceptions of emotional quality of the relationship with their child (parent-child dysfunctional interaction) and bias in parental reports as a consequence of the urge to present a favorable impression of themselves (defensive responding). Thus, this scale captures a wider perspective of parenting stress by including various components related to stress that parents can experience as a result of the demands of being a parent. Costa et al. (2006) found that parent-child dysfunctional interactions in particular were associated with child internalizing symptoms. Even though they expected specificity of associations between another parenting stress factor, the difficult child factor, and externalizing behaviors, this was not supported by their results. Children with difficult temperament and challenging behavioral characteristics can make it difficult for parents to care and manage (Abidin, 1995). Although not supported by Costa et al. (2006), the different factors within the parenting stress construct as assessed in their study may explain differences in findings between the specific relations between parenting stress and internalizing and externalizing behaviors. In our study, parenting stress was measured through the 6-item Parental Stressors Scale of the Parental PSS (Crnic et al., 2002), a shorter and less extensive measure, which may have resulted in less variation of scores over time and thereby offers a less specific presentation of the associations with child behavior outcomes. When more components within the parenting stress constructs are assessed, more variation in scores may exist across waves. Thereby, the PSI-SF may be able to disentangle the associations between the different components of parenting stress and child internalizing and externalizing behavior more specifically. To further examine the effects of parenting stress on child internalizing and externalizing behaviors during the early years of childhood, we investigated whether parenting stress assessed when the child was 9 months old was associated to child internalizing and externalizing behaviors. Parenting stress at 9 months was not associated with either child internalizing behaviors or externalizing behaviors. It is remarkable though, that parenting stress at 9 months was not predictive of parenting stress at age 3, which may suggest that early parenting stress is temporary. Our results further demonstrate that parental warmth and parental hostility did not mediate the associations between parenting stress and child internalizing and externalizing behaviors, which is not consistent with our hypotheses. Our findings are not in line with Cherry et al. (2019), who showed that observed parental supportiveness at age 2 mediated the association between parenting stress at age 1 and general child behavioral problems at age 3. Mackler et al. (2015), who measured parenting behaviors by means of a parent’s report on their own behaviors, did not find a mediating effect of negative parental reactions toward child’s behaviors on the association between parenting stress and externalizing behavior, which is consistent with our results. Several explanations can be given for the discrepancies in findings regarding mediation by parenting behaviors. A first explanation could be that Cherry et al. (2019) assessed parental behavior through observation during a parent-child play task, which is a different way of measuring parental behaviors than asking parent’s own perceptions of their behavior as we did in our study. Secondly, the constructs to measure parenting behaviors varied between the studies. Cherry et al. (2019) looked at parental supportiveness as a composite of sensitivity, positive regard and cognitive stimulation, and therefore can be regarded as a parenting behavior that is also involved in parental warmth, but is not completely similar to parental warmth. Likewise, Mackler et al. (2015) investigated parental reactions toward child’s negative behaviors, which may be related to hostile parenting behaviors. Parental reactions were assessed using self-report including responses to a child’s negative behaviors, such as punishment, distress and minimization. These behaviors have commonalities with hostile behaviors as assessed in our study, including items such as “I have raised my voice with or shouted at this child.” Nonetheless, the constructs are not completely identical, which make comparison difficult and may therefore explain discrepancies in findings between Mackler et al. (2015) and Cherry et al. (2019) and our study. A third explanation for the unexpected lack of mediating effects of parental warmth and hostility could be that the time intervals between measurements of the constructs under examination were too large to actually capture a mediating effect of the parenting behaviors. Possibly, mediation can only be measured when the mediator is more proximal in time to the variables of which it explains the association. Cherry et al. (2019) used a time lag of 2 years, and concluded mediation, whereas in Mackler et al. (2015) the lags varied between 3 and 5 years, and they did not find mediation. Thus, the total time interval of 6 years as used in the current study could have been too long to measure an actual mediation effect (Boele et al., 2020). We found that parenting stress and parental hostility, as well as parental hostility and child externalizing behaviors were highly associated cross-sectionally. Thus, it may be that a mediation effect of parental hostility might have occurred when the associations were measured closer in time. Furthermore, our null-findings regarding the mediation effect may also be due to the dimensions examined in our study. Other, maybe less specific, dimensions of parenting may better explain the associations between parenting stress and child internalizing and externalizing behaviors. For instance, authoritative parenting, defined as parents who show warmth, responsiveness, and control (Zubrick et al., 2014), is a broader measure of parenting and has been associated with internalizing behaviors (Williams et al., 2009). Another broader dimension of parenting, authoritarian parenting, characterized by parents who are low on warmth and high on restrictiveness and firm control (Zubrick et al., 2014), has been associated with externalizing behaviors. Thus, differences in conceptualization and type of measurement may explain discrepancies in findings regarding the mediation effect. Even though our results suggest that parental warmth and hostility do not explain the associations between parenting stress and child internalizing and externalizing behaviors, results show some relevant associations between parental behaviors, parenting stress and child behaviors. For instance, parenting stress at age 5 was predictive of parental warmth at age 9 when looking at parental warmth as mediator between parenting stress and internalizing behavior, after correction for covariates. Also, parenting stress at age 5 predicted later parental hostility when we tested the mediating effect of parental hostility in the association between parenting stress and externalizing behaviors. A similar association was found in the model of externalizing behavior. These findings are consistent with previous research indicating that parenting stress challenges parenting behavior, resulting in poorer parenting practices (Crnic et al., 2005; Pinquart, 2017). Furthermore, results show that child externalizing behaviors were positively associated with later parental hostility at both time points, thus indicating that in families with a child with increased levels of externalizing behaviors at ages 3 and 5 years, parents reported increased levels of later parental hostility. This is in line with Patterson’s model explaining the mutual contribution of negative parenting and child misbehavior by reinforcing one another (Patterson and Oregon, 1982). Only externalizing behaviors at age 5 were negatively associated with parental warmth at age 9, suggesting that in families with a child with increased levels of externalizing behaviors at age 5, parents reported lower levels of parental warmth. For internalizing behaviors, these associations were not found, suggesting that the coercion model by Patterson may be less or not applicable to more inward-directed behaviors. According to Patterson’s theory, coercion occurs when a person experiences the child’s behavior as aversive, which may less be the case in internalizing behaviors when compared to a child with externalizing behaviors (Patterson and Oregon, 1982). These results are of high relevance as it emphasizes the need to distinguish between internalizing and externalizing behaviors of children when examining parenting and child effects in relation to parenting stress. This study comes with several strengths. The first strength is that by the inclusion of a random-intercept to the classical CLPM, time-invariant differences between persons were decomposed from within-person changes in parenting stress and child internalizing and externalizing behaviors. We evidently showed that the more advanced models, the RI-CLPM, had a better fit than the CLPM, indicating that parenting stress, parental behaviors and child behaviors have trait-like components that should be considered in future studies. A second strength is that, as opposed to the previous studies investigating parenting stress and child behavior outcomes using cross-lagged path models (Mackler et al., 2015; Kochanova et al., 2021), our study corrected for covariates that have widely been associated with parenting stress, including household income, educational status and occupational status (Abidin, 1990, 1992). We tested models with and without covariates and demonstrated that these models were different from each other. All in all, these findings strengthen the importance of the inclusion of relevant covariates when investigating associations between parenting stress and child outcomes in order to prevent the underestimation or overestimation of the associations. Other strengths of the current study include the study population being nationally representative and including a wide range of socio-economic backgrounds, which increases the generalizability of the findings to the Irish population, and the low attrition rates across a relatively large time span of approximately 9 years. Nonetheless, the results of this study should be interpreted considering some limitations. First of all, even though we found transactional associations between parental stress, parenting behaviors, and child behaviors, the strength of the associations is rather small. Secondly, we did not control for other parental mental health outcomes, such as depression and anxiety, which have been associated with parenting stress in the literature (Patterson and Oregon, 1982; Deater-Deckard, 1998; Jennings and Dietz, 2007). It may not be surprising that, when parents experience high levels of depressive or anxiety symptoms, they may find parenting challenging, resulting in parenting stress. Mental health constructs, such as stress, depression, and anxiety may co-occur, however, have clear distinctions with regard to their origin, biological responses, and expressions (Abidin, 1992). When taking into account all interrelated constructs of parenting stress, we would not examine the bidirectional associations of parenting stress as a complete construct, but would investigate solely the variance of parenting stress that is left after controlling for other mental health constructs. Therefore, our study examined parenting stress in relation to child outcomes without taking into account the possible interrelated variables such as depression and anxiety. A third limitation is that parenting stress, parental behaviors, and child behavior outcomes were all reported by primary caregivers of the children. Parental reports on child’s internalizing behaviors are commonly found to be underreported, for instance as a consequence of internalizing symptoms being less visible to parents (Tandon et al., 2009). As a result of this possible underreporting of internalizing behaviors, parents might also experience less stress. Bias might also have occurred as parents who experience higher parenting stress reporting more negatively on their child’s internalizing and externalizing behaviors than the actual behaviors that the child performs. Even though they studied an older sample than we did in our study, a large longitudinal study by Robinson et al. (2019), comparing adolescent’s own externalizing behaviors with parental reports, found that agreement in scores was impacted most strongly when parents scored higher on parental depression, stress, and family dysfunction. This finding implies that highly stressed parents in particular may report more externalizing behaviors when compared to parents that are less stressed. Shared methods variance may also play a role when parents rate their own parenting behaviors, and can have resulted in an overestimation of the actual effect as parents with higher levels of stress are more likely to report more negatively on their own behaviors. Reports of secondary caregivers or teachers could have been included to account for bias in parental and child behavior reports. Secondary caregivers and teachers may have a different experience about the child’s behavior than the primary caregiver (Kraemer et al., 2003; de Maat et al., 2021), as they might observe the child’s behaviors in other contexts. A fourth limitation is that all outcomes are based on questionnaire data only. A multimethod approach could be applied, by combining both questionnaires and observational data to obtain a more objective measure of parental and child behavior (de Maat et al., 2021). By combining observational measures with questionnaires, single rater bias can be investigated and possibly reduced. Also, in two-parent families, crossover effects may play a role. It is likely that in a household with two caregivers, one caregiver could affect the behavior of the other (de Maat et al., 2021). de Maat et al. (2021) investigated associations between parenting stress, problem behavior, and parenting behaviors in adolescents, and looked at possible crossover effects of mothers and fathers. They found that when fathers reported higher levels of parenting stress, mothers performed more maladaptive parenting behaviors, and vice versa. A crossover effect might also result in a compensation effect, such that parental reports on own scores are reduced by the views or experiences of the secondary caregiver. Thus, future studies may investigate parenting and child outcomes from a more family systems perspective by including other family members, including other children in the household (Richmond et al., 2005), as well. A final limitation to mention is that we were unable to investigate full bidirectionality from age 9 months to 9 years, due to missing information about child behavior when the child was 9 months old. Our results of the sensitivity analyses did, however, clearly demonstrate how additional information in such an already complex model can change the results. Thus, it is recommended for future studies to investigate reciprocal relations between parenting stress and child behaviors, starting as early as possible to investigate how early child and parent factors may develop and influence one another across childhood. In conclusion, our results show that parenting stress is an antecedent and a consequence of child behavior, for child internalizing and externalizing behaviors separately, from age 5 to 9 but not before age 5, although a child effect from externalizing behavior to parenting stress was found from age 3 to 5. Our results did not indicate mediating effects of parental warmth or parental hostility in the associations between parenting stress and child behavior outcomes. However, future research should further investigate mediation by including more proximal time points, or even including highly frequent longitudinal data collection using Experienced Based Sampling (Bolger and Laurenceau, 2013) techniques. Mediating mechanisms could also be further examined by including other parenting dimensions (e.g., authoritarian parenting), other methods (e.g., observations), and different informants (e.g., secondary caregiver). The field of parenting and child behaviors needs further exploration using transactional models considering the complex methodological approaches and family dynamics. As it is clear that associations are not unidirectional *per se*, and that developmental processes including various child and family factors play a role, clinical practice should consider a broad and systemic approach. In such an approach, families with parents who experience parenting stress and children with dysfunctional behaviors will be involved as a whole when developing or performing interventions. Families may benefit from early intervention in order to prevent escalation involving complex reciprocal effects of both the children and their parents.

## Data availability statement

The data analyzed in this study is subject to the following licenses/restrictions: Data can be accessed on application to the Irish Social Science Data Archive. Requests to access these datasets should be directed to www.ucd.ie/issda.

## Ethics statement

Ethical approval was granted by a dedicated Research Ethics Committee established by the Department of Health and Children of Ireland. Written informed consent to participate in this study was provided by the participants’ legal guardian/next of kin.

## Author contributions

AH, MO, and KM-S: review and editing. MM: methodology, analysis, and review and editing. WVD: analysis, writing—original draft, and visualization. All authors contributed to the article and approved the submitted version.

## Footnotes

1
http://www.esri.ie/growing-up-in-ireland/about-growing-up-in-ireland/
