# Corrigendum: Longitudinal relations between parenting stress and child internalizing and externalizing behaviors: testing within-person changes, bidirectionality and mediating mechanisms

**DOI:** 10.3389/fnbeh.2023.1296453

**Published:** 2023-11-07

**Authors:** Willeke Van Dijk, Marleen H. M. de Moor, Mirjam Oosterman, Anja C. Huizink, Karen Matvienko-Sikar

**Affiliations:** ^1^Department of Clinical, Neuro and Developmental Psychology, Amsterdam Public Health Research Institute, Vrije Universiteit Amsterdam, Amsterdam, Netherlands; ^2^Department of Psychology, Education and Child Studies, Erasmus School of Social and Behavioural Sciences, Erasmus University Rotterdam, Rotterdam, Netherlands; ^3^Department of Clinical Child and Family Studies, Amsterdam Public Health Research Institute, Vrije Universiteit Amsterdam, Amsterdam, Netherlands; ^4^School of Public Health, University College Cork, Cork, Ireland

**Keywords:** parenting behavior, child behavioral problems, bidirectional relations, random intercept cross-lagged panel model, parenting stress

In the published article, the citation for an author name was incorrectly written as Dijk et al. (2022). The correct spelling is Van Dijk et al. (2022).

In the published article, the contributions of the author was reported using the initials WD. The correct use of the initials is WVD.

In the published article, the reference for “*Parenting stress* can be assessed using a variety of measures, with some scales consisting only of items asking about parent domains, such as the Parenting Stress Scale (PSS)” was incorrectly written as (Crnic et al., 2002). It should be (Berry and Jones, 1995).

In the published article, the reference for “and others comprising child domains as well, including the Parenting Stress Index (PSI)” was incorrectly written as (Deater-Deckard and Panneton, 2017). It should be (Abidin, 1995).

In the published article, the reference for “And the Parenting Daily Hassles scale (PDH);” was incorrectly written as (Patterson, 1992). It should be (Crnic and Greenberg, 1990). The full citation for this reference is: Crnic, K. A., and Greenberg, M. T. (1990). Minor parenting stresses with young children. *Child Dev*. 61, 1628–1637.

In the published article, the reference for [“…in their systematic review that parental warmth was negatively associated with behavioral outcomes in preterm children, as displayed by fewer internalizing and externalizing problems.] was incorrectly written as Baumrind (1971). It should be Neel et al. (2018).

In the published article, the reference for “Families with children registered on the Child Benefit Register born between December 1st 2007 and June 30th 2008 could be included, with a total of 41,185 eligible children” was incorrectly written as (Rogosa, 1980). It should be (Quail et al., 2011).

In the published article, the reference for “Children's emotional and behavioral problems were assessed by the Strengths and Difficulties Questionnaire (SDQ);” was incorrectly written as (Hamaker et al., 2015). It should be (Goodman, 1997).

In the published article, the reference for “For the current study, the scores on the subscale Emotional Symptoms and Peer Problems were combined by summarizing the scores to create an “Internalizing” subscale, and the Conduct Problems and Hyperactivity subscale were combined by summarizing the scores to create an “Externalizing” subscale” was incorrectly written as (Quail et al., 2011). It should be (Goodman et al., 2010).

In the published article, the reference for “The subscales demonstrated acceptable reliability (Internalizing Cronbach's a = 0.73, Externalizing Cronbach's a = 0.78;” was incorrectly written as (Goodman, 1997). It should be (Goodman et al., 2010).

In the published article, the reference for “Parenting behavior was measured using an instrument from the Longitudinal Study of Australian Children (LSAC);” was incorrectly written as (Anthony et al., 2005). It should be (Zubrick et al., 2014).

In the published article, the reference for “To investigate the within-person associations of parenting stress and child internalizing and externalizing behavior problems over time, random-intercept crossed lagged panel modeling (RI-CLPM) were used in R using the Rstudio software v.1.2.5019 and the Lavaan package” was incorrectly written as (Goodman, 1997). It should be (Rosseel, 2012).

In the published article, the reference for “Primary caregiver's cultural background and child's gender were included in the model as TIC's, and primary caregiver's age, partner status, household income, occupational status, and educational level were included in the model as TVC's” was incorrectly written as (Goodman et al., 2010). It should be (Mund et al., 2021).

In the published article, the reference for “the Tucker–Lewis Index (TLI;” was incorrectly written as Rosseel, 2012. It should be (Tucker and Lewis, 1973).

In the published article, the reference for “A maximum likelihood estimator (ML) with robust standard errors was used to handle missing values and adjust for any deviations from normality” was incorrectly written as (Hu and Bentler, 1998). It should be (Enders, 2001).

In the published article, the reference for “As opposed to externalizing behaviors, internalizing behaviors could easily go unnoticed by parents, and maybe also because at very young ages children's verbal skills are less well-developed yet, which makes them less competent in expressing their internal feelings” was incorrectly written as (Kline, 2015). It should be (Tandon et al., 2009).

In the published article, the reference for “This idea is also supported by studies examining correspondence between different informants, with often larger correspondence found in reports of children's externalizing problems when compared to internalizing problems] was incorrectly written as (Enders, 2001; Tandon et al., 2009”. It should be (van der Ende et al., 2012; De Los Reyes et al., 2015).

In the published article, the reference for [“… investigated the specificity of parenting stressors and disentangled the associations between various parenting stressors and internalizing and externalizing behaviors,…] was incorrectly written as van der Ende et al. (2012). It should be Costa et al. (2006).

In the published article, the reference for “(PSI-SF;” was incorrectly written as (Abidin, 1995). It should be (Abidin, 1990).

In the published article, the reference for “found that parent-child dysfunctional interactions in particular were associated with child internalizing symptoms” was incorrectly written as van der Ende et al. (2012). It should be Costa et al. (2006).

In the published article, the reference for “Children with difficult temperament and challenging behavioral characteristics can make it difficult for parents to care and manage] was incorrectly written as (De Los Reyes et al., 2015)”. It should be (Abidin, 1995).

In the published article, the reference for “Although not supported by” was incorrectly written as van der Ende et al. (2012). It should be Costa et al. (2006).

In the published article, the reference for “Thus, the total time interval of 6 years as used in the current study could have been too long to measure an actual mediation effect” was incorrectly written as (Costa et al., 2006). It should be (Boele et al., 2020).

In the published article, the reference for “our study corrected for covariates that have widely been associated with parenting stress, including household income, educational status and occupational status” was incorrectly written as (Abidin, 1990, 1995). It should be (Abidin, 1990, 1992).

In the published article, the reference for “Parental reports on child's internalizing behaviors are commonly found to be underreported, for instance as a consequence of internalizing symptoms being less visible to parents” was incorrectly written as (Kline, 2015). It should be (Tandon et al., 2009).

In the published article, the reference for “Even though they studied an older sample than we did in our study, a large longitudinal study by” was incorrectly written as Boele et al. (2020). It should be Robinson et al. (2019).

In the published article, the reference for “Secondary caregivers and teachers may have a different experience about the child's behavior than the primary caregiver” was incorrectly written as (Bradley and Corwyn, 2002; de Maat et al., 2021). It should be (Kraemer et al., 2003; de Maat et al., 2021).

In the published article, the reference for “A multimethod approach could be applied, by combining both questionnaires and observational data to obtain a more objective measure of parental and child behavior” was incorrectly written as (Tandon et al., 2009). It should be (de Maat et al., 2021).

In the published article, the reference for “Thus, future studies may investigate parenting and child outcomes from a more family systems perspective by including other family members, including other children in the household” was incorrectly written as (Richmond et al., 2005; Robinson et al., 2019). It should be (Richmond et al., 2005).

In the published article, the reference for “However, future research should further investigate mediation by including more proximal time points, or even including highly frequent longitudinal data collection using Experienced Based Sampling” was incorrectly written as (Kraemer et al., 2003; Bolger and Laurenceau, 2013). It should be (Bolger and Laurenceau, 2013).

The authors apologize for this error and state that this does not change the scientific conclusions of the article in any way. The original article has been updated.
