# Supplementary material for: Longitudinal relations between parenting stress and child internalizing and externalizing behaviors: Testing within-person changes, bidirectionality and mediating mechanisms
Source: Front Behav Neurosci. 2022 Dec 16;16:942363. doi: 10.3389/fnbeh.2022.942363 (PMC9800797; doi:10.3389/fnbeh.2022.942363)
Supplement: Supplementary file 1 [file Table_1.docx]

**Supplementary Table 1.**

*Differences in Parenting Stress Between the Covariates*

|  | Wave 1 |  |  | Wave 2 |  |  | Wave 3 |  |  | Wave 5 |  |  |
| --- | --- | --- | --- | --- | --- | --- | --- | --- | --- | --- | --- | --- |
|  | M *(SD)* | *t/F* | *p* | M *(SD)* | *t* | *p* | M *(SD)* | *t* | *p* | M *(SD)* | *t* | *p* |
| Gender child  Girls    Boys | 14.41 (4.19)  14.65 (4.14) | 2.41 | .02 | 12.10 (4.09)  12.36 (4.10) | 2.66 | <.01 | 11.57 (3.88)  11.77 (4.15) | 2.19 | .03 | 13.23 (4.31)  13.59 (4.46) | 3.41 | <.001 |
| Cultural background  White  Non-White | 14.45 (4.11)  16.39 (4.76) | -7.31 | <.001 | 12.17 (4.06)  13.43 (4.70) | -4.69 | <.001 | 11.62 (3.98)  12.63 (4.63) | -3.86 | <.001 | 13.39 (4.37)  13.89 (4.95) | -1.75 | .08 |
| Partnered  Yes    No | 14.41 (4.08)  15.77 (4.78) | 6.98 | <.001 | 12.06 (4.00)  13.72 (4.57) | 9.46 | <.001 | 11.47 (3.91)  13.34 (4.53) | 10.91 | <.001 | 13.31 (4.33)  14.25 (4.78) | 5.19 | <.001 |
| Education  Up to third  Third or  higher | 14.58 (4.29)  14.46 (3.98) | 1.18 | .24 | 12.15 (4.22)  12.34 (3.90) | -1.96 | .05 | 11.56 (4.09)  11.84 (3.91) | -2.84 | <.01 | 13.16 (4.44)  13.79 (4.30) | -5.91 | <.001 |
| Occupation  Not employed  Employed | 14.97 (4.40)  14.27 (4.00) | 6.77 | <.001 | 12.39 (4.29)  12.12 (3.96) | 2.61 | <.01 | 11.74 (4.27)  11.62 (3.84) | 1.26 | .21 | 13.46 (4.66)  13.38 (4.26) | .65 | .52 |
| Age PC  Young  Old | 14.60 (4.27)  14.50 (4.13) | 0.80 | .42 | 12.93 (4.36)  12.10 (4.03) | 6.00 | <.001 | 12.50 (4.26)  11.58 (3.98) | 5.49 | <.001 | 13.60 (4.42)  13.31 (4.37) | 2.65 | <.01 |
| Household income  Q1    Q2    Q3    Q4    Q5 | 15.29 (4.54)  14.66 (4.29)  14.52 (4.26)  14.25 (3.94)  14.08 (3.84) | 15.98 | <.001 | 12.48 (4.37)  12.46 (4.29)  12.09 (4.12)  12.22 (4.07)  12.04 (3.73) | 3.27 | .01 | 11.94 (4.38)  11.77 (4.13)  11.63 (3.99)  11.49 (3.84)  11.64 (3.82) | 2.28 | .06 | 13.40 (4.52)  13.33 (4.57)  13.38 (4.38)  13.70 (4.28)  13.38 (4.12) | 1.67 | .16 |

*Note.* PC = Primary Caregiver; M (SD) = mean (standard deviation); Q1 = 1^st^ quintile; Q2 = 2^nd^ quintile; Q3 = 3^rd^ quintile; Q4 = 4^th^ quintile; Q5 = 5^th^ quintile.
