# Supplementary material for: Longitudinal relations between parenting stress and child internalizing and externalizing behaviors: Testing within-person changes, bidirectionality and mediating mechanisms
Source: Front Behav Neurosci. 2022 Dec 16;16:942363. doi: 10.3389/fnbeh.2022.942363 (PMC9800797; doi:10.3389/fnbeh.2022.942363)
Supplement: Supplementary file 2 [file Table_2.docx]

**Supplementary Table 2.**

*Differences in Child Internalizing Behavior Between the Covariates*

|  | Wave 2 |  |  | Wave 3 |  |  | Wave 5 |  |  |
| --- | --- | --- | --- | --- | --- | --- | --- | --- | --- |
|  | M *(SD)* | *t* | *p* | M *(SD)* | *t* | *p* | M *(SD)* | *t* | *p* |
| Gender child  Girls    Boys | 2.37 (2.09)  2.53 (2.22) | 2.99 | <.01 | 2.42 (2.32)  2.49 (2.48) | 1.28 | .20 | 2.94 (2.78)  2.99 (2.99) | .82 | .41 |
| Cultural background  White  Non-White | 2.42 (2.14)  3.02 (2.51) | -4.25 | <.001 | 2.42 (2.38)  3.05 (2.71) | -4.18 | <.001 | 2.97 (2.90)  2.88 (2.59) | .58 | .57 |
| Partnered  Yes    No | 2.39 (2.13)  2.97 (2.38) | 6.36 | <.001 | 2.37 (2.33)  3.16 (2.80) | 7.56 | <.001 | 2.86 (2.81)  3.84 (3.37) | 7.79 | <.001 |
| Education PC  Up to third  Third or  Higher | 2.59 (2.21)  2.22 (2.06) | 7.21 | <.001 | 2.60 (2.49)  2.21 (2.23) | 6.89 | <.001 | 3.20 (3.01)  2.65 (2.65) | 8.88 | <.001 |
| Occupation PC  Not employed  Employed | 2.66 (2.33)  2.31 (2.03) | 6.62 | <.001 | 2.72 (2.57)  2.28 (2.26) | 7.49 | <.001 | 3.36 (3.13)  2.79 (2.76) | 7.46 | <.001 |
| Age PC  Young  Old | 2.98 (2.39)  2.35 (2.10) | 8.45 | <.001 | 3.21 (2.73)  2.37 (2.35) | 7.83 | <.001 | 3.39 (3.10)  2.74 (2.74) | 8.86 | <.001 |
| Household income  Q1    Q2    Q3    Q4    Q5 | 2.84 (2.34)  2.75 (2.29)  2.59 (2.20)  2.21 (2.03)  2.05 (1.92) | 36.49 | <.001 | 2.87 (2.65)  2.68 (2.52)  2.47 (2.32)  2.28 (2.30)  2.14 (2.24) | 20.82 | <.001 | 3.43 (3.12)  3.32 (3.08)  3.02 (2.94)  2.74 (2.76)  2.58 (2.62) | 20.43 | <.001 |

*Note.* PC = Primary Caregiver; M (SD) = mean (standard deviation); Q1 = 1^st^ quintile; Q2 = 2^nd^ quintile; Q3 = 3^rd^ quintile; Q4 = 4^th^ quintile; Q5 = 5^th^ quintile.
