# Supplementary material for: Longitudinal relations between parenting stress and child internalizing and externalizing behaviors: Testing within-person changes, bidirectionality and mediating mechanisms
Source: Front Behav Neurosci. 2022 Dec 16;16:942363. doi: 10.3389/fnbeh.2022.942363 (PMC9800797; doi:10.3389/fnbeh.2022.942363)
Supplement: Supplementary file 3 [file Table_3.docx]

**Supplementary Table 3.**

*Differences in Child Externalizing Behavior Between the Covariates*

|  | Wave 2 |  |  | Wave 3 |  |  | Wave 5 |  |  |
| --- | --- | --- | --- | --- | --- | --- | --- | --- | --- |
|  | M *(SD)* | *t* | *p* | M *(SD)* | *t* | *p* | M *(SD)* | *t* | *p* |
| Gender child  Girls    Boys | 4.86 (3.19)  5.42 (3.33) | 7.35 | <.001 | 4.21 (3.11)  5.09 (3.47) | 11.42 | <.001 | 3.54 (3.07)  4.75 (3.59) | 15.38 | <.001 |
| Cultural background  White  Non-White | 5.13 (3.28)  5.36 (3.21) | -1.28 | .20 | 4.67 (3.32)  4.31 (3.25) | 1.92 | .06 | 4.17 (3.42)  3.67 (2.81) | 3.16 | <.01 |
| Partnered  Yes    No | 2.86 (2.81)  3.84 (3.37) | 7.79 | <.001 | 4.99 (3.20)  6.40 (3.65) | 10.10 | <.001 | 4.51 (3.24)  5.80 (3.71) | 9.21 | <.001 |
| Education PC  Up to third  Third or  higher | 5.48 (3.38)  4.59 (3.04) | 11.59 | <.001 | 4.97 (3.43)  4.15 (3.08) | 10.51 | <.001 | 4.45 (3.52)  3.68 (3.13) | 9.60 | <.001 |
| Occupation PC  Not employed  Employed | 5.42 (3.39)  4.95 (3.18) | 6.00 | <.001 | 5.01 (3.52)  4.41 (3.16) | 7.33 | <.001 | 4.52 (3.60)  3.98 (3.29) | 6.04 | <.001 |
| Age PC  Young  Old | 6.11 (4.46)  4.95 (3.21) | 10.61 | <.001 | 5.98 (3.61)  4.51 (3.26) | 10.37 | <.001 | 4.64 (3.52)  3.89 (3.30) | 8.92 | <.001 |
| Household income  Q1    Q2    Q3    Q4    Q5 | 5.73 (3.45)  5.74 (3.45)  5.34 (3.28)  4.80 (3.10)  4.46 (3.03) | 43.38 | <.001 | 5.25 (3.61)  5.15 (3.48)  4.70 (3.24)  4.35 (3.14)  4.09 (3.08) | 31.79 | <.001 | 4.81 (3.74)  4.58 (3.61)  4.01 (3.29)  3.89 (3.21)  3.71 (3.15) | 23.77 | <.001 |

*Note.* PC = Primary Caregiver; M (SD) = mean (standard deviation); Q1 = 1^st^ quintile; Q2 = 2^nd^ quintile; Q3 = 3^rd^ quintile; Q4 = 4^th^ quintile; Q5 = 5^th^ quintile.
