# Supplementary material for: Longitudinal relations between parenting stress and child internalizing and externalizing behaviors: Testing within-person changes, bidirectionality and mediating mechanisms
Source: Front Behav Neurosci. 2022 Dec 16;16:942363. doi: 10.3389/fnbeh.2022.942363 (PMC9800797; doi:10.3389/fnbeh.2022.942363)
Supplement: Supplementary file 4 [file Table_4.docx]

**Supplementary Table 4.**

*Differences in Parental Warmth Between the Covariates*

|  | Wave 2 |  |  | Wave 3 |  |  | Wave 5 |  |  |
| --- | --- | --- | --- | --- | --- | --- | --- | --- | --- |
|  | M *(SD)* | *t* | *p* | M *(SD)* | *t* | *p* | M *(SD)* | *t* | *p* |
| Gender child  Girls    Boys | 4.75 (0.37)  4.74 (0.37) | -.89 | .38 | 4.73 (0.40)  4.72 (0.41) | -.45 | .66 | 4.54 (0.57)  4.52 (0.58) | -1.56 | .12 |
| Cultural background  White  Non-White | 4.74 (0.37)  4.70 (0.44) | -1.68 | .09 | 4.73 (0.40)  4.70 (0.45) | 1.07 | .29 | 4.53 (0.57)  4.47 (0.62) | 1.84 | .07 |
| Partnered  Yes    No | 4.74 (0.37)  4.77 (0.37) | 1.84 | .07 | 4.74 (0.41)  4.72 (0.40) | 1.15 | .25 | 4.52 (0.58)  4.59 (0.54) | 3.09 | <.01 |
| Education PC  Up to third  Third or  higher | 4.75 (0.37)  4.72 (0.38) | 4.25 | <.001 | 4.73 (0.40)  4.71 (0.40) | 1.99 | .05 | 4.55 (0.57)  4.50 (0.59) | 3.59 | <.001 |
| Occupation PC  Not employed  Employed | 4.73 (0.39)  4.75 (0.36) | -2.86 | <.01 | 4.72 (0.40)  4.73 (0.40) | -1.07 | .28 | 4.52 (0.59)  4.53 (0.57) | -0.55 | .59 |
| Age  Young  Old | 4.76 (0.37)  4.74 (0.37) | 1.35 | .18 | 4.73 (0.40)  4.73 (0.40) | 0.33 | .74 | 4.50 (0.59)  4.54 (0.57) | -2.74 | <.01 |
| Household income  Q1    Q2    Q3    Q4    Q5 | 4.75 (0.38)  4.74 (0.39)  4.73 (0.38)  4.73 (0.37)  4.76 (0.36) | 1.27 | .28 | 4.71 (0.41)  4.71 (0.42)  4.73 (0.39)  4.73 (0.40)  4.73 (0.39) | 0.80 | .53 | 4.50 (0.60)  4.52 (0.58)  4.54 (0.54)  4.51 (0.59)  4.55 (0.57) | 1.82 | .12 |

*Note.* PC = Primary Caregiver; M (SD) = mean (standard deviation); Q1 = 1^st^ quintile; Q2 = 2^nd^ quintile; Q3 = 3^rd^ quintile; Q4 = 4^th^ quintile; Q5 = 5^th^ quintile.
