# Supplementary material for: Longitudinal relations between parenting stress and child internalizing and externalizing behaviors: Testing within-person changes, bidirectionality and mediating mechanisms
Source: Front Behav Neurosci. 2022 Dec 16;16:942363. doi: 10.3389/fnbeh.2022.942363 (PMC9800797; doi:10.3389/fnbeh.2022.942363)
Supplement: Supplementary file 5 [file Table_5.docx]

**Supplementary Table 5.**

*Differences in Parental Hostility Between the Covariates*

|  | Wave 2 |  |  | Wave 3 |  |  | Wave 5 |  |  |
| --- | --- | --- | --- | --- | --- | --- | --- | --- | --- |
|  | M *(SD)* | *t* | *p* | M *(SD)* | *t* | *p* | M *(SD)* | *t* | *p* |
| Gender child  Girls    Boys | 1.77 (0.48)  1.81 (0.49) | 3.03 | <.01 | 1.78 (0.49)  1.82 (0.50) | 3.92 | <.001 | 1.98 (0.61)  2.05 (0.61) | 4.95 | <.001 |
| Cultural background  White  Non-White | 1.79 (0.48)  1.86 (0.54) | -2.38 | .02 | 1.80 (0.49)  1.80 (0.51) | -0.10 | .92 | 2.02 (0.61)  2.06 (0.62) | -1.06 | .29 |
| Partnered  Yes    No | 1.79 (0.48)  1.80 (0.50) | 0.58 | .56 | 1.80 (0.49)  1.82 (0.54) | 0.68 | .50 | 2.02 (0.61)  2.01 (0.64) | -0.47 | .64 |
| Education PC  Up to third  Third or  higher | 1.79 (0.49)  1.80 (0.47) | -1.05 | .29 | 1.78 (0.50)  1.83 (0.49) | -4.22 | <.001 | 2.01 (0.62)  2.04 (0.60) | -2.44 | .02 |
| Occupation PC  Not employed  Employed | 1.79 (0.50)  1.79 (0.48) | 0.29 | .77 | 1.79 (0.50)  1.81 (0.49) | -2.01 | .05 | 2.02 (0.62)  2.02 (0.61) | 0.22 | .83 |
| Age  Young  Old | 1.81 (0.48)  1.79 (0.48) | 1.01 | .31 | 1.79 (0.52)  1.80 (0.49) | -0.58 | .56 | 2.08 (0.62)  1.99 (0.61) | 5.75 | <.001 |
| Household income  Q1    Q2    Q3    Q4    Q5 | 1.78 (0.50)  1.80 (0.49)  1.82 (0.49)  1.81 (0.48)  1.77 (0.46) | 2.63 | 0.03 | 1.78 (0.52)  1.78 (0.49)  1.81 (0.49)  1.82 (0.48)  1.82 (0.50) | 2.10 | .08 | 2.00 (0.64)  2.02 (0.60)  2.02 (0.61)  2.06 (0.62)  2.01 (0.59) | 1.63 | .16 |

*Note.* PC = Primary Caregiver; M (SD) = mean (standard deviation); Q1 = 1^st^ quintile; Q2 = 2^nd^ quintile; Q3 = 3^rd^ quintile; Q4 = 4^th^ quintile; Q5 = 5^th^ quintile
