# Supplementary material for: Longitudinal relations between parenting stress and child internalizing and externalizing behaviors: Testing within-person changes, bidirectionality and mediating mechanisms
Source: Front Behav Neurosci. 2022 Dec 16;16:942363. doi: 10.3389/fnbeh.2022.942363 (PMC9800797; doi:10.3389/fnbeh.2022.942363)
Supplement: Supplementary file 6 [file Table_6.docx]

Supplementary Material

**Supplementary Table 6.**

*Pearson Product-Moment Correlations Between the Parenting Stress, Parental Warmth, Parenting Hostility, Child Internalizing Behavior and Child Externalizing Behavior at Waves 1, 2, 3, and 5.*

|  | 1 | 2 | 3 | 4 | 5 | 6 | 7 | 8 | 9 | 10 | 11 | 12 | 13 | 14 | 15 |
| --- | --- | --- | --- | --- | --- | --- | --- | --- | --- | --- | --- | --- | --- | --- | --- |
| 1.PSS W2 | - |  |  |  |  |  |  |  |  |  |  |  |  |  |  |
| 2.PSS W3 | .58** | - |  |  |  |  |  |  |  |  |  |  |  |  |  |
| 3.PSS W5 | .47** | .52** | - |  |  |  |  |  |  |  |  |  |  |  |  |
| 4.Warmth W2 | -.16** | -.15** | -.12** | - |  |  |  |  |  |  |  |  |  |  |  |
| 5.Warmth W3 | -.13** | -.18** | -.13** | .43** | - |  |  |  |  |  |  |  |  |  |  |
| 6.Warmth W5 | -.13** | -.15** | -.24** | .32** | .38** | - |  |  |  |  |  |  |  |  |  |
| 7.Hostility W2 | .35** | .28** | .24** | -.29** | -.23** | -.21** | - |  |  |  |  |  |  |  |  |
| 8.Hostility W3 | .28** | .37** | .27** | -.21** | -.32** | -.23** | .49** | - |  |  |  |  |  |  |  |
| 9.Hostility W5 | .24** | .28** | .38** | -.15** | -.18** | -.37** | .38** | .43** | - |  |  |  |  |  |  |
| 10.INT W2 | .27** | .23** | .17** | -.12** | -.11** | -.10** | .24** | .16** | .15** | - |  |  |  |  |  |
| 11.INT W3 | .24** | .33** | .21** | -.08** | -.11** | -.09** | .18** | .21** | .14** | .45** | - |  |  |  |  |
| 12.INT W5 | .23** | .28** | .30** | -.04** | -.06** | -.10** | .16** | .18** | .20** | .34** | .47** | - |  |  |  |
| 13.EXT W2 | .35** | .28** | .24****** | -.17** | -.13** | -.12** | .46** | .33** | .27** | .31** | .25** | .27** | - |  |  |
| 14.EXT W3 | .28** | .38** | .27** | -.11** | -.17** | -.15** | .31** | .44** | .30** | .23** | .34** | .31** | .55** | - |  |
| 15.EXT W5 | .25** | .32** | .34** | -.09** | -.10** | -.20** | .22** | .30** | .40** | .19** | .26** | .43** | .44** | .60** | - |

*Note.* **Correlation is significant at the 0.01 level (2- tailed). W1 = Wave 1; W2 = Wave 2; W3 = Wave 3; W5 = Wave 5; PSS=Parenting Stress Scale; INT = Internalizing behavior; EXT = Externalizing behavior.

**Supplementary Table 6.**

*Differences in Parenting Stress Between the Covariates*

|  | Wave 1 |  |  | Wave 2 |  |  | Wave 3 |  |  | Wave 5 |  |  |
| --- | --- | --- | --- | --- | --- | --- | --- | --- | --- | --- | --- | --- |
|  | M *(SD)* | *t/F* | *p* | M *(SD)* | *t* | *p* | M *(SD)* | *t* | *p* | M *(SD)* | *t* | *p* |
| Gender child  Girls    Boys | 14.41 (4.19)  14.65 (4.14) | 2.41 | .02 | 12.10 (4.09)  12.36 (4.10) | 2.66 | <.01 | 11.57 (3.88)  11.77 (4.15) | 2.19 | .03 | 13.23 (4.31)  13.59 (4.46) | 3.41 | <.001 |
| Cultural background  White  Non-White | 14.45 (4.11)  16.39 (4.76) | -7.31 | <.001 | 12.17 (4.06)  13.43 (4.70) | -4.69 | <.001 | 11.62 (3.98)  12.63 (4.63) | -3.86 | <.001 | 13.39 (4.37)  13.89 (4.95) | -1.75 | .08 |
| Partnered  Yes    No | 14.41 (4.08)  15.77 (4.78) | 6.98 | <.001 | 12.06 (4.00)  13.72 (4.57) | 9.46 | <.001 | 11.47 (3.91)  13.34 (4.53) | 10.91 | <.001 | 13.31 (4.33)  14.25 (4.78) | 5.19 | <.001 |
| Education  Up to third  Third or  higher | 14.58 (4.29)  14.46 (3.98) | 1.18 | .24 | 12.15 (4.22)  12.34 (3.90) | -1.96 | .05 | 11.56 (4.09)  11.84 (3.91) | -2.84 | <.01 | 13.16 (4.44)  13.79 (4.30) | -5.91 | <.001 |
| Occupation  Not employed  Employed | 14.97 (4.40)  14.27 (4.00) | 6.77 | <.001 | 12.39 (4.29)  12.12 (3.96) | 2.61 | <.01 | 11.74 (4.27)  11.62 (3.84) | 1.26 | .21 | 13.46 (4.66)  13.38 (4.26) | .65 | .52 |
| Age PC  Young  Old | 14.60 (4.27)  14.50 (4.13) | 0.80 | .42 | 12.93 (4.36)  12.10 (4.03) | 6.00 | <.001 | 12.50 (4.26)  11.58 (3.98) | 5.49 | <.001 | 13.60 (4.42)  13.31 (4.37) | 2.65 | <.01 |
| Household income  Q1    Q2    Q3    Q4    Q5 | 15.29 (4.54)  14.66 (4.29)  14.52 (4.26)  14.25 (3.94)  14.08 (3.84) | 15.98 | <.001 | 12.48 (4.37)  12.46 (4.29)  12.09 (4.12)  12.22 (4.07)  12.04 (3.73) | 3.27 | .01 | 11.94 (4.38)  11.77 (4.13)  11.63 (3.99)  11.49 (3.84)  11.64 (3.82) | 2.28 | .06 | 13.40 (4.52)  13.33 (4.57)  13.38 (4.38)  13.70 (4.28)  13.38 (4.12) | 1.67 | .16 |

*Note.* PC = Primary Caregiver; M (SD) = mean (standard deviation); Q1 = 1^st^ quintile; Q2 = 2^nd^ quintile; Q3 = 3^rd^ quintile; Q4 = 4^th^ quintile; Q5 = 5^th^ quintile.

**Supplementary Table 7.**

*Differences in Child Internalizing Behavior Between the Covariates*

|  | Wave 2 |  |  | Wave 3 |  |  | Wave 5 |  |  |
| --- | --- | --- | --- | --- | --- | --- | --- | --- | --- |
|  | M *(SD)* | *t* | *p* | M *(SD)* | *t* | *p* | M *(SD)* | *t* | *p* |
| Gender child  Girls    Boys | 2.37 (2.09)  2.53 (2.22) | 2.99 | <.01 | 2.42 (2.32)  2.49 (2.48) | 1.28 | .20 | 2.94 (2.78)  2.99 (2.99) | .82 | .41 |
| Cultural background  White  Non-White | 2.42 (2.14)  3.02 (2.51) | -4.25 | <.001 | 2.42 (2.38)  3.05 (2.71) | -4.18 | <.001 | 2.97 (2.90)  2.88 (2.59) | .58 | .57 |
| Partnered  Yes    No | 2.39 (2.13)  2.97 (2.38) | 6.36 | <.001 | 2.37 (2.33)  3.16 (2.80) | 7.56 | <.001 | 2.86 (2.81)  3.84 (3.37) | 7.79 | <.001 |
| Education PC  Up to third  Third or  Higher | 2.59 (2.21)  2.22 (2.06) | 7.21 | <.001 | 2.60 (2.49)  2.21 (2.23) | 6.89 | <.001 | 3.20 (3.01)  2.65 (2.65) | 8.88 | <.001 |
| Occupation PC  Not employed  Employed | 2.66 (2.33)  2.31 (2.03) | 6.62 | <.001 | 2.72 (2.57)  2.28 (2.26) | 7.49 | <.001 | 3.36 (3.13)  2.79 (2.76) | 7.46 | <.001 |
| Age PC  Young  Old | 2.98 (2.39)  2.35 (2.10) | 8.45 | <.001 | 3.21 (2.73)  2.37 (2.35) | 7.83 | <.001 | 3.39 (3.10)  2.74 (2.74) | 8.86 | <.001 |
| Household income  Q1    Q2    Q3    Q4    Q5 | 2.84 (2.34)  2.75 (2.29)  2.59 (2.20)  2.21 (2.03)  2.05 (1.92) | 36.49 | <.001 | 2.87 (2.65)  2.68 (2.52)  2.47 (2.32)  2.28 (2.30)  2.14 (2.24) | 20.82 | <.001 | 3.43 (3.12)  3.32 (3.08)  3.02 (2.94)  2.74 (2.76)  2.58 (2.62) | 20.43 | <.001 |

*Note.* PC = Primary Caregiver; M (SD) = mean (standard deviation); Q1 = 1^st^ quintile; Q2 = 2^nd^ quintile; Q3 = 3^rd^ quintile; Q4 = 4^th^ quintile; Q5 = 5^th^ quintile.

**Supplementary Table 8.**

*Differences in Child Externalizing Behavior Between the Covariates*

|  | Wave 2 |  |  | Wave 3 |  |  | Wave 5 |  |  |
| --- | --- | --- | --- | --- | --- | --- | --- | --- | --- |
|  | M *(SD)* | *t* | *p* | M *(SD)* | *t* | *p* | M *(SD)* | *t* | *p* |
| Gender child  Girls    Boys | 4.86 (3.19)  5.42 (3.33) | 7.35 | <.001 | 4.21 (3.11)  5.09 (3.47) | 11.42 | <.001 | 3.54 (3.07)  4.75 (3.59) | 15.38 | <.001 |
| Cultural background  White  Non-White | 5.13 (3.28)  5.36 (3.21) | -1.28 | .20 | 4.67 (3.32)  4.31 (3.25) | 1.92 | .06 | 4.17 (3.42)  3.67 (2.81) | 3.16 | <.01 |
| Partnered  Yes    No | 2.86 (2.81)  3.84 (3.37) | 7.79 | <.001 | 4.99 (3.20)  6.40 (3.65) | 10.10 | <.001 | 4.51 (3.24)  5.80 (3.71) | 9.21 | <.001 |
| Education PC  Up to third  Third or  higher | 5.48 (3.38)  4.59 (3.04) | 11.59 | <.001 | 4.97 (3.43)  4.15 (3.08) | 10.51 | <.001 | 4.45 (3.52)  3.68 (3.13) | 9.60 | <.001 |
| Occupation PC  Not employed  Employed | 5.42 (3.39)  4.95 (3.18) | 6.00 | <.001 | 5.01 (3.52)  4.41 (3.16) | 7.33 | <.001 | 4.52 (3.60)  3.98 (3.29) | 6.04 | <.001 |
| Age PC  Young  Old | 6.11 (4.46)  4.95 (3.21) | 10.61 | <.001 | 5.98 (3.61)  4.51 (3.26) | 10.37 | <.001 | 4.64 (3.52)  3.89 (3.30) | 8.92 | <.001 |
| Household income  Q1    Q2    Q3    Q4    Q5 | 5.73 (3.45)  5.74 (3.45)  5.34 (3.28)  4.80 (3.10)  4.46 (3.03) | 43.38 | <.001 | 5.25 (3.61)  5.15 (3.48)  4.70 (3.24)  4.35 (3.14)  4.09 (3.08) | 31.79 | <.001 | 4.81 (3.74)  4.58 (3.61)  4.01 (3.29)  3.89 (3.21)  3.71 (3.15) | 23.77 | <.001 |

*Note.* PC = Primary Caregiver; M (SD) = mean (standard deviation); Q1 = 1^st^ quintile; Q2 = 2^nd^ quintile; Q3 = 3^rd^ quintile; Q4 = 4^th^ quintile; Q5 = 5^th^ quintile.

**Supplementary Table 9.**

*Differences in Parental Warmth Between the Covariates*

|  | Wave 2 |  |  | Wave 3 |  |  | Wave 5 |  |  |
| --- | --- | --- | --- | --- | --- | --- | --- | --- | --- |
|  | M *(SD)* | *t* | *p* | M *(SD)* | *t* | *p* | M *(SD)* | *t* | *p* |
| Gender child  Girls    Boys | 4.75 (0.37)  4.74 (0.37) | -.89 | .38 | 4.73 (0.40)  4.72 (0.41) | -.45 | .66 | 4.54 (0.57)  4.52 (0.58) | -1.56 | .12 |
| Cultural background  White  Non-White | 4.74 (0.37)  4.70 (0.44) | -1.68 | .09 | 4.73 (0.40)  4.70 (0.45) | 1.07 | .29 | 4.53 (0.57)  4.47 (0.62) | 1.84 | .07 |
| Partnered  Yes    No | 4.74 (0.37)  4.77 (0.37) | 1.84 | .07 | 4.74 (0.41)  4.72 (0.40) | 1.15 | .25 | 4.52 (0.58)  4.59 (0.54) | 3.09 | <.01 |
| Education PC  Up to third  Third or  higher | 4.75 (0.37)  4.72 (0.38) | 4.25 | <.001 | 4.73 (0.40)  4.71 (0.40) | 1.99 | .05 | 4.55 (0.57)  4.50 (0.59) | 3.59 | <.001 |
| Occupation PC  Not employed  Employed | 4.73 (0.39)  4.75 (0.36) | -2.86 | <.01 | 4.72 (0.40)  4.73 (0.40) | -1.07 | .28 | 4.52 (0.59)  4.53 (0.57) | -0.55 | .59 |
| Age  Young  Old | 4.76 (0.37)  4.74 (0.37) | 1.35 | .18 | 4.73 (0.40)  4.73 (0.40) | 0.33 | .74 | 4.50 (0.59)  4.54 (0.57) | -2.74 | <.01 |
| Household income  Q1    Q2    Q3    Q4    Q5 | 4.75 (0.38)  4.74 (0.39)  4.73 (0.38)  4.73 (0.37)  4.76 (0.36) | 1.27 | .28 | 4.71 (0.41)  4.71 (0.42)  4.73 (0.39)  4.73 (0.40)  4.73 (0.39) | 0.80 | .53 | 4.50 (0.60)  4.52 (0.58)  4.54 (0.54)  4.51 (0.59)  4.55 (0.57) | 1.82 | .12 |

*Note.* PC = Primary Caregiver; M (SD) = mean (standard deviation); Q1 = 1^st^ quintile; Q2 = 2^nd^ quintile; Q3 = 3^rd^ quintile; Q4 = 4^th^ quintile; Q5 = 5^th^ quintile.

**Supplementary Table 10.**

*Differences in Parental Hostility Between the Covariates*

|  | Wave 2 |  |  | Wave 3 |  |  | Wave 5 |  |  |
| --- | --- | --- | --- | --- | --- | --- | --- | --- | --- |
|  | M *(SD)* | *t* | *p* | M *(SD)* | *t* | *p* | M *(SD)* | *t* | *p* |
| Gender child  Girls    Boys | 1.77 (0.48)  1.81 (0.49) | 3.03 | <.01 | 1.78 (0.49)  1.82 (0.50) | 3.92 | <.001 | 1.98 (0.61)  2.05 (0.61) | 4.95 | <.001 |
| Cultural background  White  Non-White | 1.79 (0.48)  1.86 (0.54) | -2.38 | .02 | 1.80 (0.49)  1.80 (0.51) | -0.10 | .92 | 2.02 (0.61)  2.06 (0.62) | -1.06 | .29 |
| Partnered  Yes    No | 1.79 (0.48)  1.80 (0.50) | 0.58 | .56 | 1.80 (0.49)  1.82 (0.54) | 0.68 | .50 | 2.02 (0.61)  2.01 (0.64) | -0.47 | .64 |
| Education PC  Up to third  Third or  higher | 1.79 (0.49)  1.80 (0.47) | -1.05 | .29 | 1.78 (0.50)  1.83 (0.49) | -4.22 | <.001 | 2.01 (0.62)  2.04 (0.60) | -2.44 | .02 |
| Occupation PC  Not employed  Employed | 1.79 (0.50)  1.79 (0.48) | 0.29 | .77 | 1.79 (0.50)  1.81 (0.49) | -2.01 | .05 | 2.02 (0.62)  2.02 (0.61) | 0.22 | .83 |
| Age  Young  Old | 1.81 (0.48)  1.79 (0.48) | 1.01 | .31 | 1.79 (0.52)  1.80 (0.49) | -0.58 | .56 | 2.08 (0.62)  1.99 (0.61) | 5.75 | <.001 |
| Household income  Q1    Q2    Q3    Q4    Q5 | 1.78 (0.50)  1.80 (0.49)  1.82 (0.49)  1.81 (0.48)  1.77 (0.46) | 2.63 | 0.03 | 1.78 (0.52)  1.78 (0.49)  1.81 (0.49)  1.82 (0.48)  1.82 (0.50) | 2.10 | .08 | 2.00 (0.64)  2.02 (0.60)  2.02 (0.61)  2.06 (0.62)  2.01 (0.59) | 1.63 | .16 |

*Note.* PC = Primary Caregiver; M (SD) = mean (standard deviation); Q1 = 1^st^ quintile; Q2 = 2^nd^ quintile; Q3 = 3^rd^ quintile; Q4 = 4^th^ quintile; Q5 = 5^th^ quintile.

**Supplementary Figure 7**

*Random Intercept Cross-Lagged Panel Model Showing Relations Among Parenting Stress (STRESS) and Child Internalizing Behaviors (INT) Across Four Waves*

**
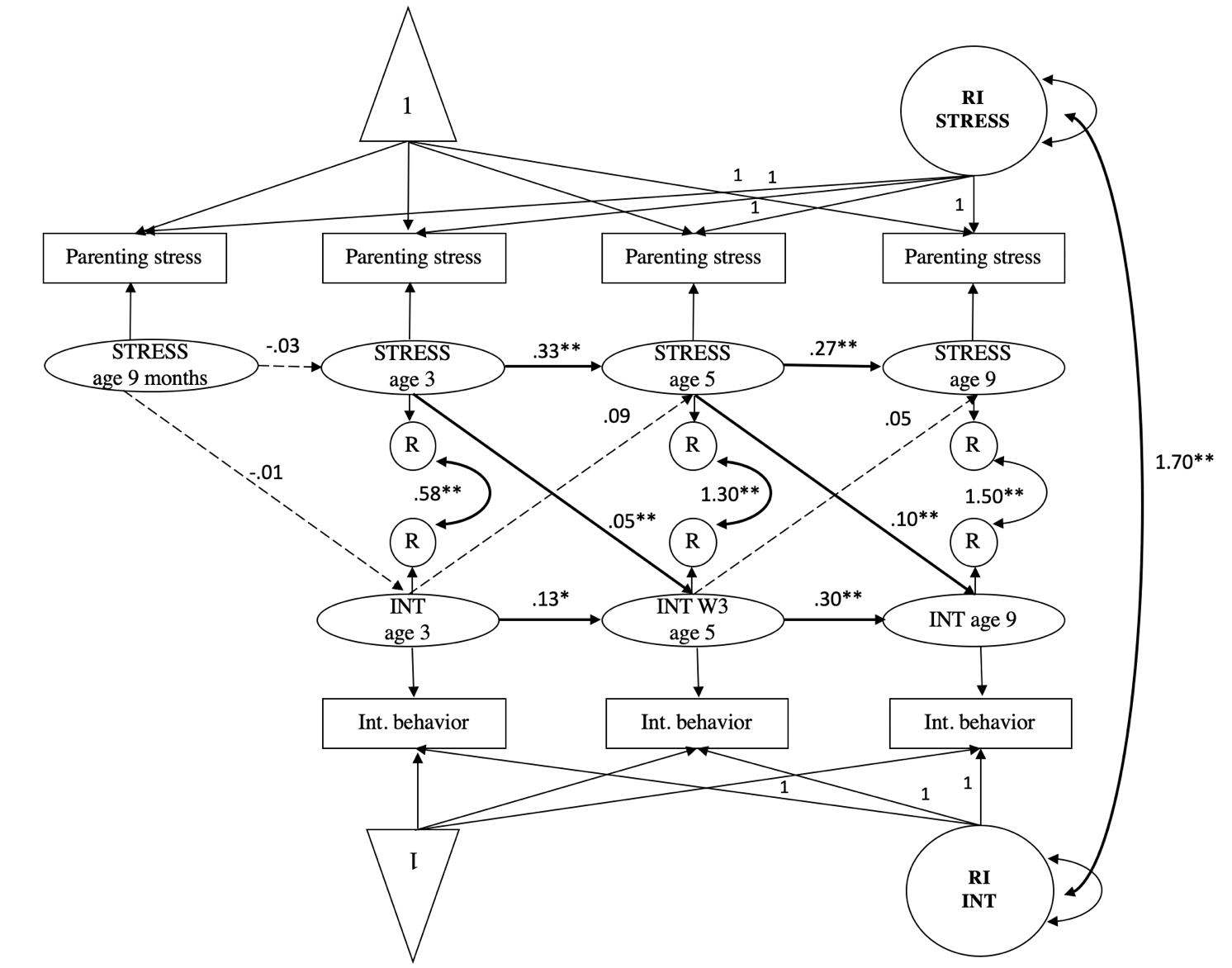
**

*Notes.* Age 9 months = wave 1; Age 3 = wave 2; age 5 = wave 3; age 9 = wave 5; R = residual variance; RI INT = random intercept child internalizing behavior; RI STRESS = random intercept parenting stress. Standardized estimates reported. Gray dashed paths indicated nonsignificant estimates. **p* < .01, ***p* < .001

**Supplementary Figure 8**

*Random Intercept Cross-Lagged Panel Model Showing Relations Among Parenting Stress (STRESS) and Child Externalizing Behaviors (EXT) Across Four Waves*


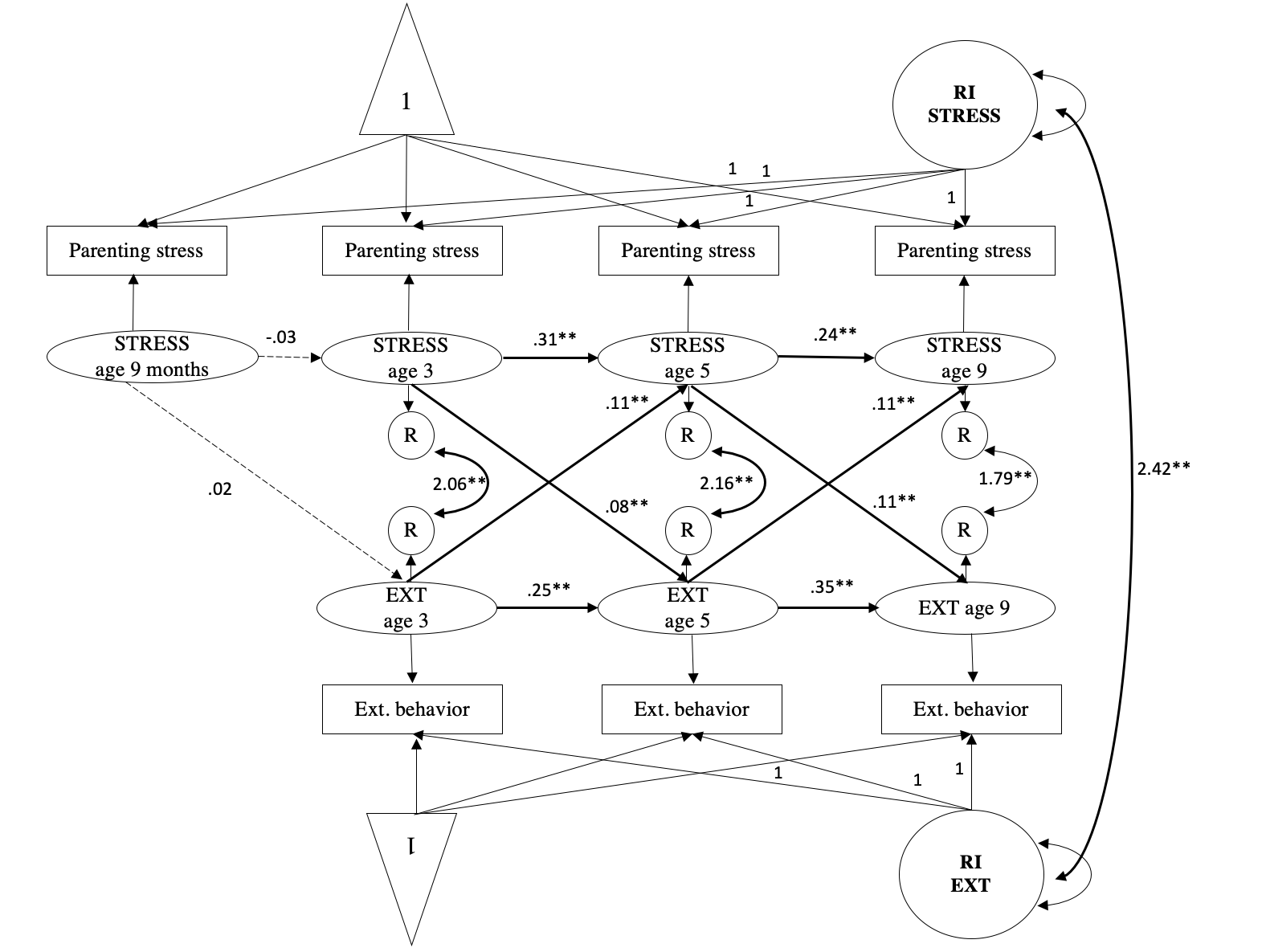


*Notes.* Age 9 months = wave 1; Age 3 = wave 2; age 5 = wave 3; age 9 = wave 5; R = residual variance; RI EXT = random intercept child externalizing behavior; RI STRESS = random intercept parenting stress. Standardized estimates reported. Gray dashed paths indicated nonsignificant estimates. **p* < .01, ***p* < .001

**Supplementary Figure 9**

*Random Intercept Cross-Lagged Panel Model Showing Relations Among Parenting Stress (STRESS) and Child Internalizing Behaviors (INT) Across Three Waves Corrected for Covariates*

**
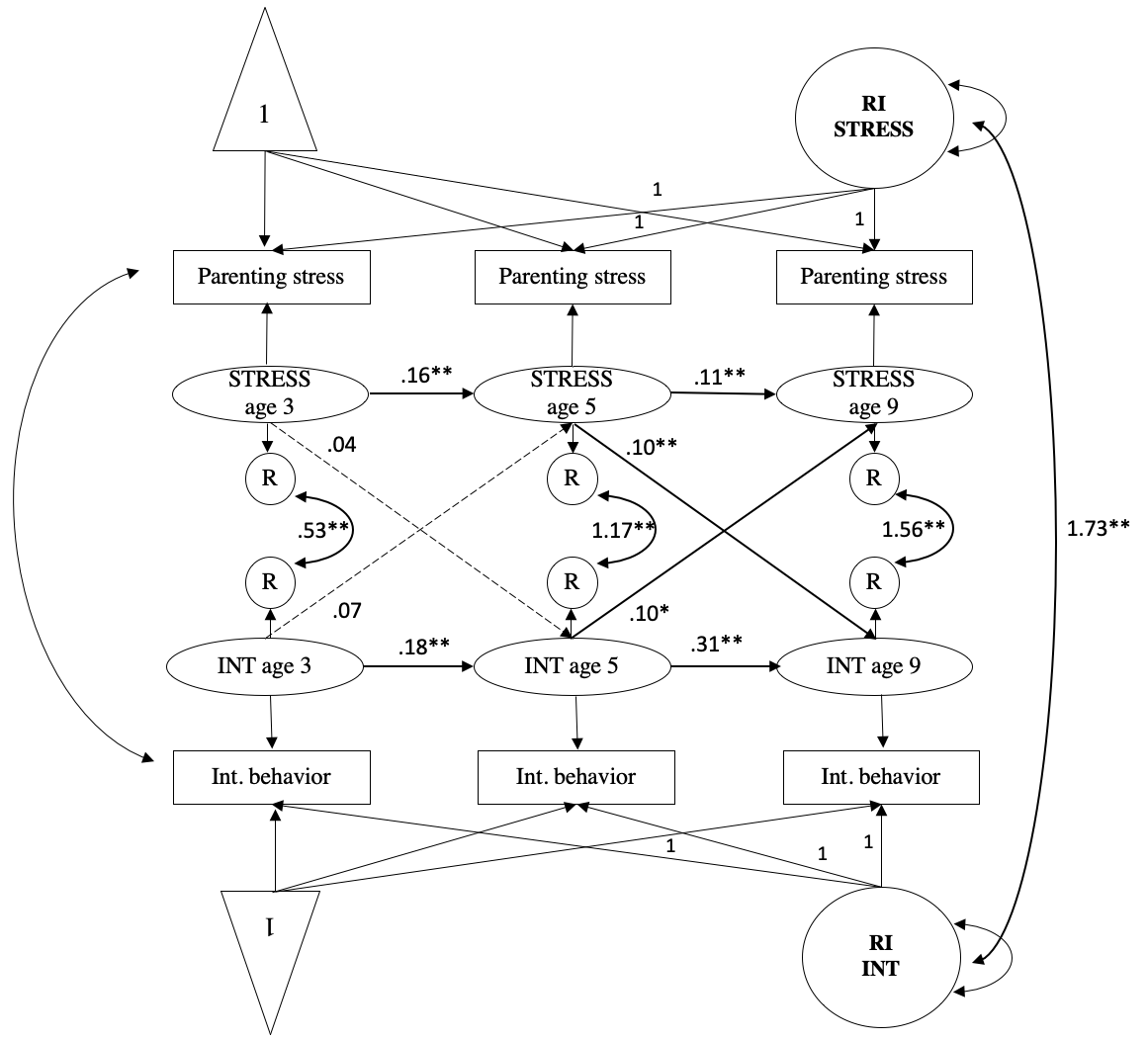
**

*Notes.* Age 3 = wave 2; age 5 = wave 3; age 9 = wave 5; R = residual variance; RI INT = random intercept child internalizing behavior; RI STRESS = random intercept parenting stress. Standardized estimates reported. Gray dashed paths indicated nonsignificant estimates. **p* < .01, ** *p* < .001

**Supplementary Figure 10**

*Random Intercept Cross-Lagged Panel Model Showing Relations Among Parenting Stress (STRESS), Parental Warmth (WARMTH) and Child Internalizing Behaviors (INT) Across Three Waves Corrected for Covariates*


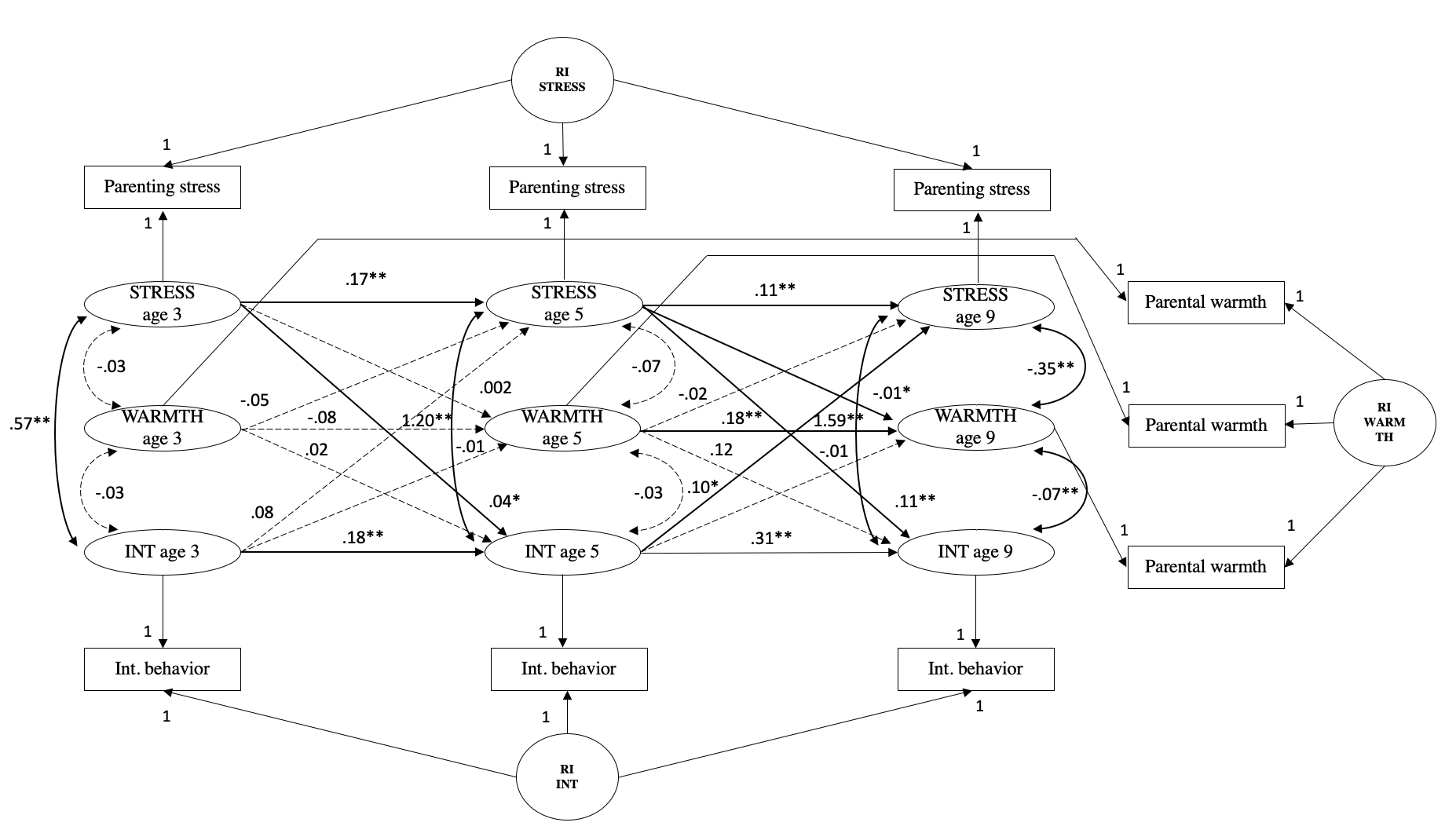


*Notes.* Age 3 = wave 2; age 5 = wave 3; age 9 = wave 5; R, residual variance = RI INT, random intercept child internalizing behavior; RI WARMTH = random intercept parental warmth; RI STRESS = random intercept parenting stress. Standardized estimates reported. Gray dashed paths indicated nonsignificant estimates. **p* < .01, ***p* < .001

**Supplementary Figure 11**

*Random Intercept Cross-Lagged Panel Model Showing Relations Among Parenting Stress (STRESS), Parental Hostility (HOST) and Child Internalizing Behaviors (INT) Across Three Waves Corrected for Covariates*


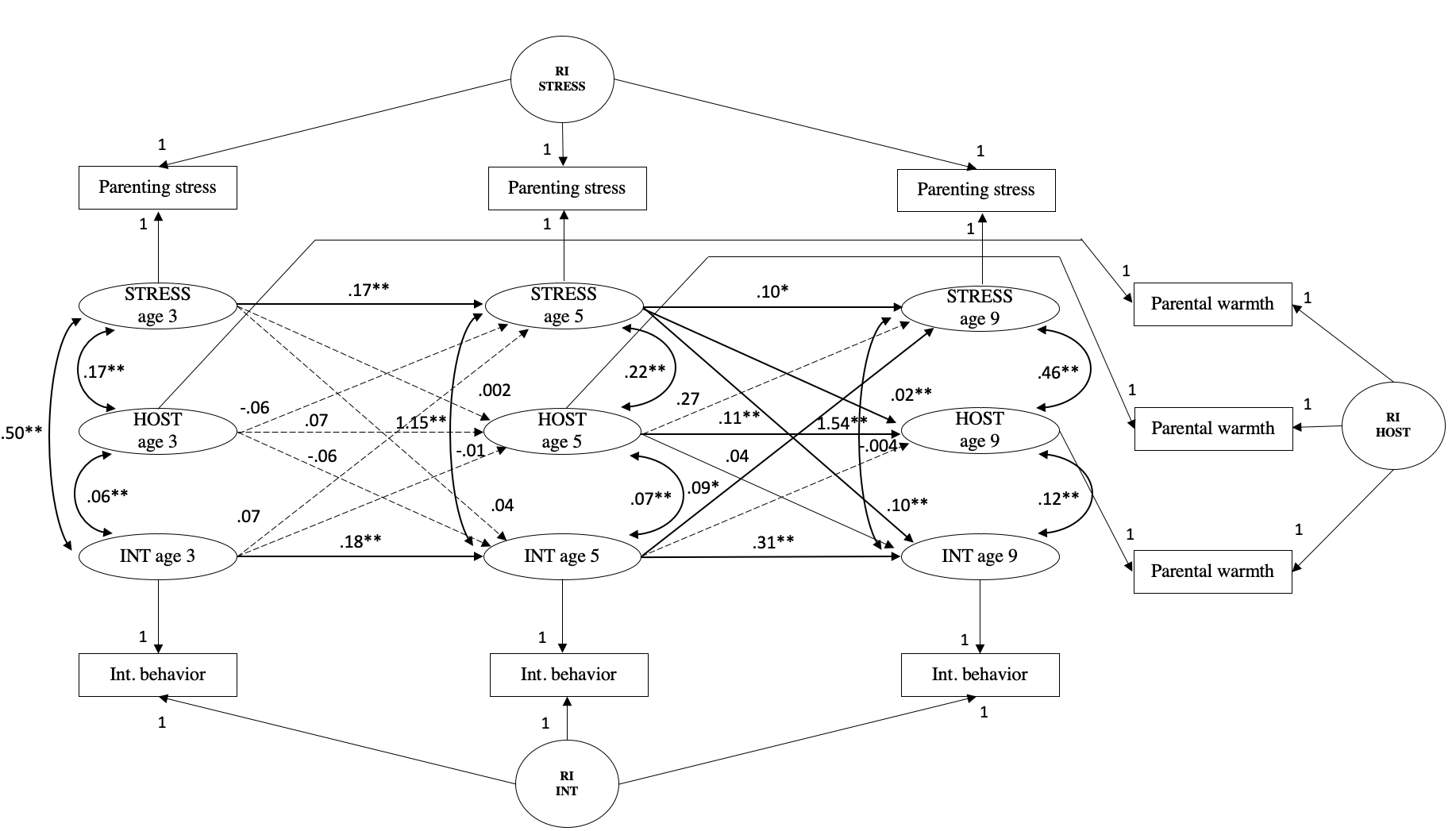


*Notes.* Age 3 = wave 2; age 5 = wave 3; age 9 = wave 5; R = residual variance; RI INT = random intercept child internalizing behavior; RI STRESS = random intercept parenting stress; RI HOST = random intercept parental hostility. Standardized estimates reported. Gray dashed paths indicated nonsignificant estimates. **p* < .01, ***p* < .001

**Supplementary Figure 12**

*Random Intercept Cross-Lagged Panel Model Showing Relations Among Parenting Stress (STRESS) and Child Externalizing Behaviors (EXT) Across Three Waves Corrected for Covariates*


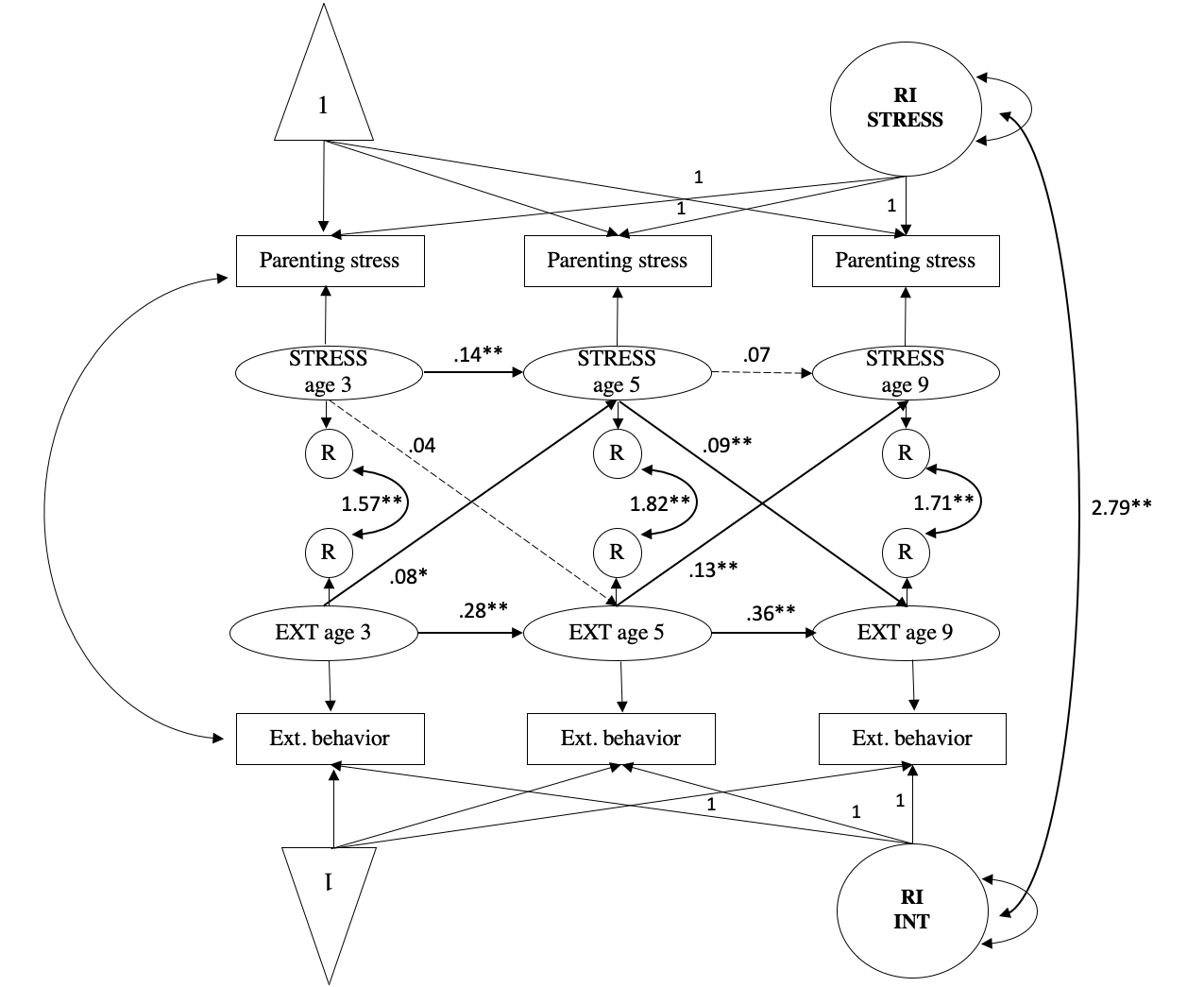


*Notes.* Age 3 = wave 2; age 5 = wave 3; age 9 = wave 5; R = residual variance; RI EXT = random intercept child externalizing behavior; RI STRESS = random intercept parenting stress. *Note.* Standardized estimates reported. Gray dashed paths indicated nonsignificant estimates. **p* < .01, ***p* < .001.

**Supplementary Figure 13**

*Random Intercept Cross-Lagged Panel Model Showing Relations Among Parenting Stress (STRESS), Parental Warmth (WARMTH) and Child Externalizing Behaviors (EXT) Across Three Waves Corrected for Covariates*


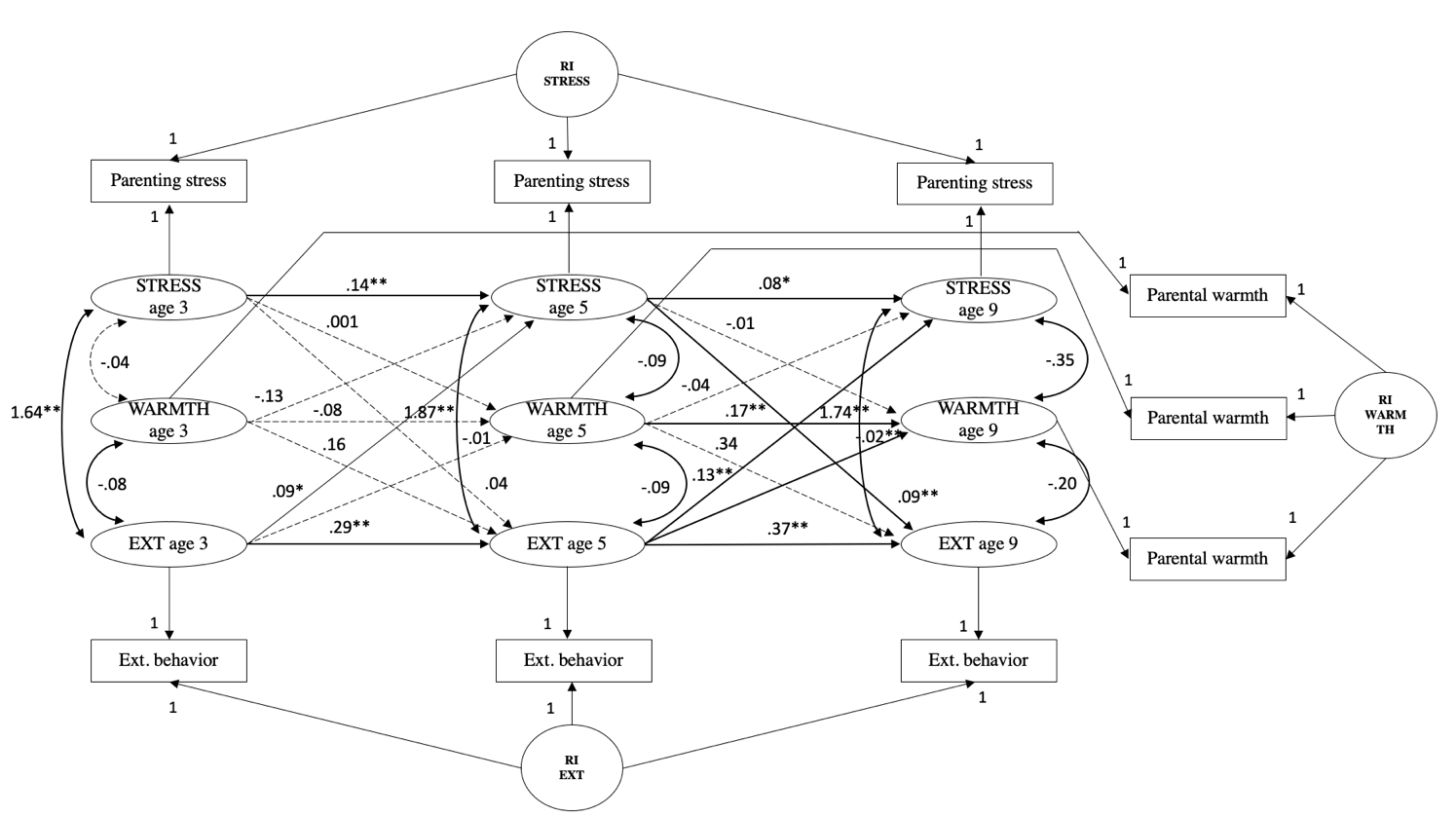


*Notes.* Age 3 = wave 2; age 5 = wave 3; age 9 = wave 5; R = residual variance; RI EXT = random intercept child externalizing behavior; RI STRESS = random intercept parenting stress; RI WARMTH = random intercept parental warmth. Standardized estimates reported. Gray dashed paths indicated nonsignificant estimates. **p* < .01, ***p* < .001

**Supplementary Figure 14**

*Random Intercept Cross-Lagged Panel Model Showing Relations Among Parenting Stress (STRESS), Parental hostility (HOST) and Child Externalizing Behaviors (EXT) Across Three Waves After Correction for Covariates*


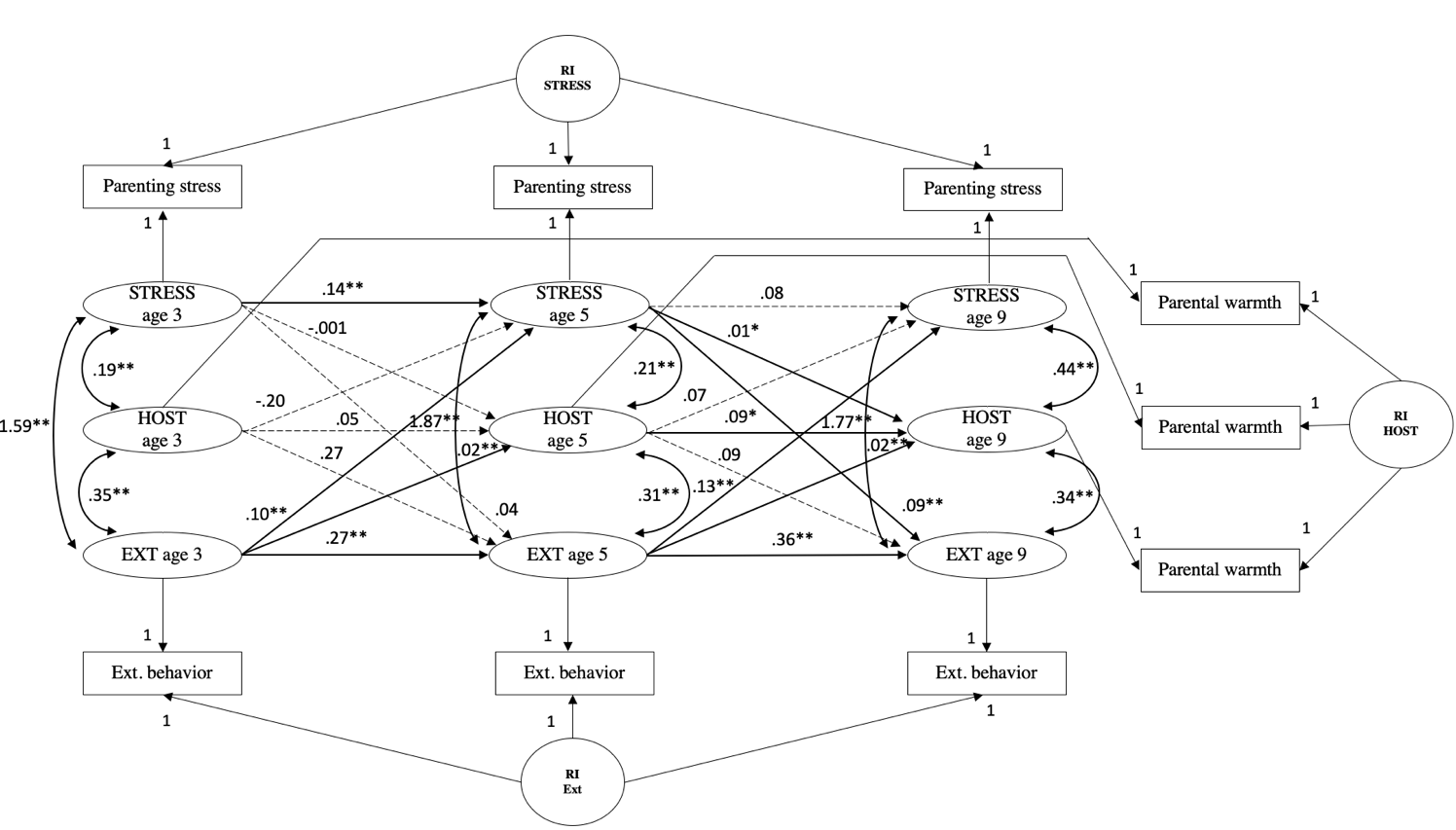


*Notes.* Age 3 = wave 2; age 5 = wave 3; age 9 = wave 5; R, residual variance; RI EXT = random intercept child externalizing behavior; RI STRESS = random intercept parenting stress; RI HOST = random intercept parental hostility. Standardized estimates reported. Gray dashed paths indicated nonsignificant estimates. **p* < .01, ***p* < .001

**Supplementary Figure 15**

*Random Intercept Cross-Lagged Panel Model Showing Relations Among Parenting Stress (STRESS) and Child Internalizing Behaviors (INT) Across Four waves, After Correction for Covariates*


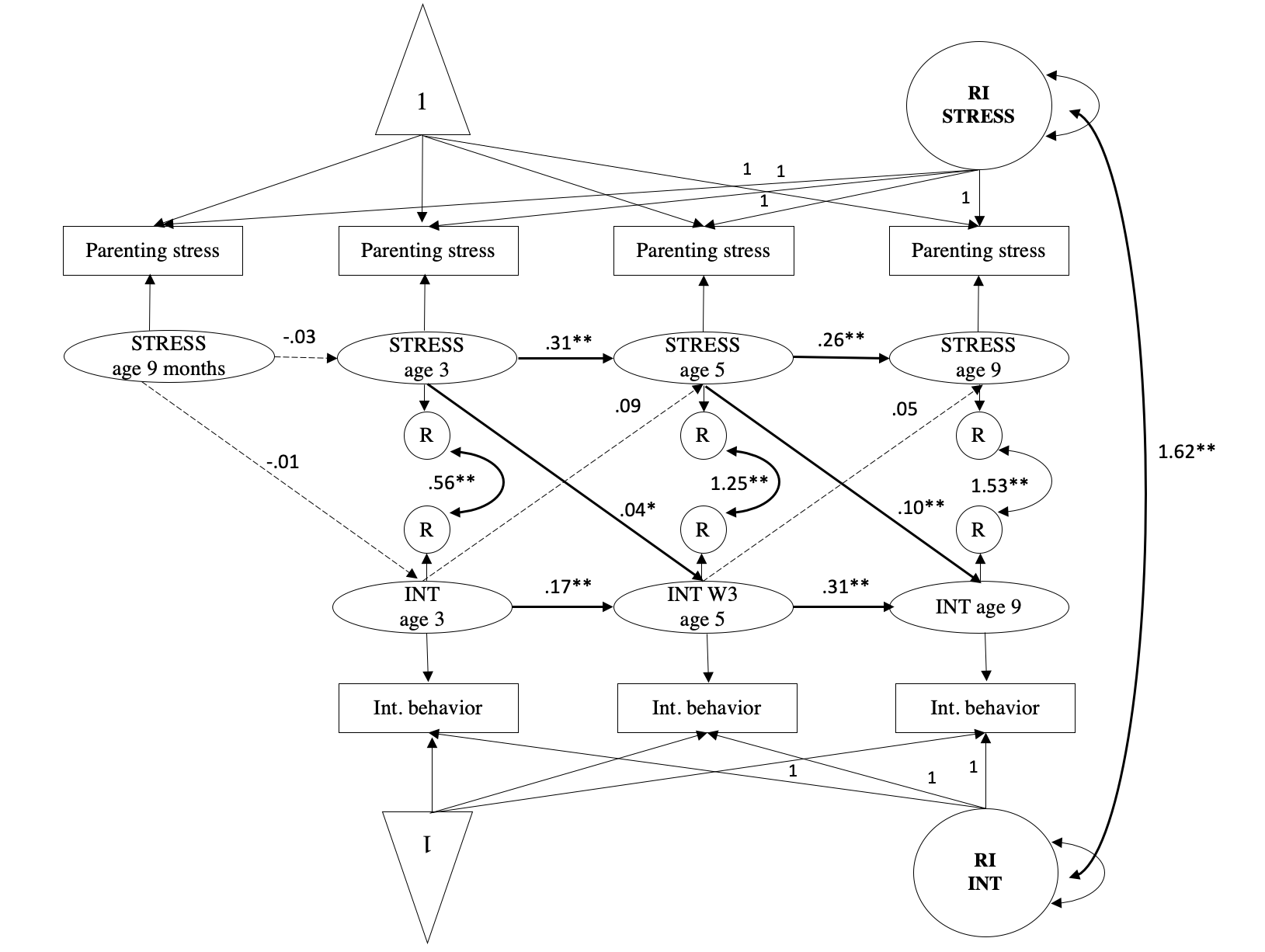


*Notes.* Age 9 months = wave 1; Age 3 = wave 2; age 5 = wave 3; age 9 = wave 5; R = residual variance; RI INT = random intercept child internalizing behavior; RI STRESS = random intercept parenting stress. Standardized estimates reported. Gray dashed paths indicated nonsignificant estimates. **p* < .01, ***p* < .001

**Supplementary Figure 16**

*Random Intercept Cross-Lagged Panel Model Showing Relations Among Parenting Stress (STRESS) and Child Externalizing Behaviors (EXT) Across Four waves, After Correction for Covariates*

**
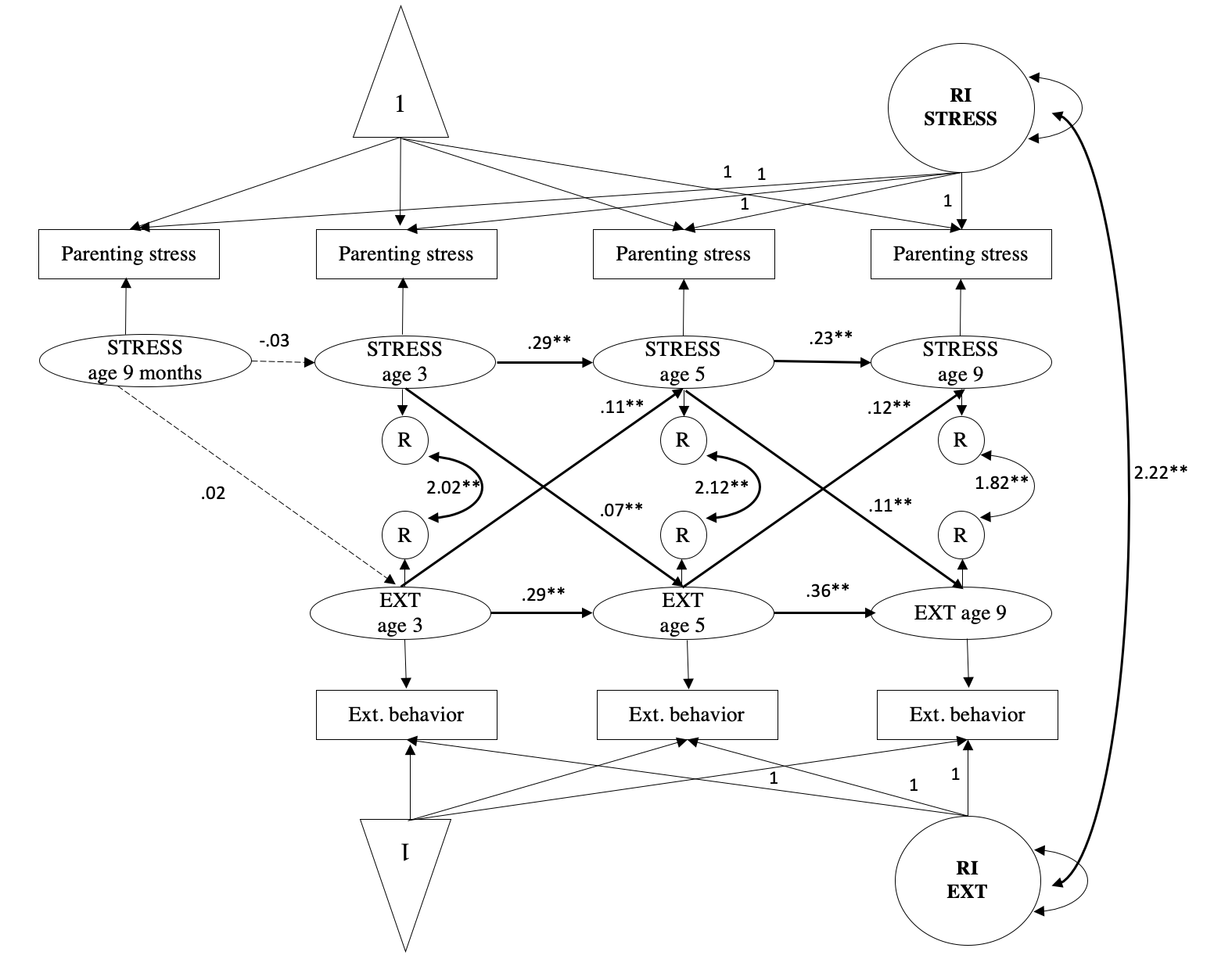
**

*Notes.* Age 9 months = wave 1; Age 3 = wave 2; age 5 = wave 3; age 9 = wave 5; R = residual variance; RI EXT = random intercept child externalizing behavior; RI STRESS = random intercept parenting stress. Standardized estimates reported. Gray dashed paths indicated nonsignificant estimates. **p* < .01, ***p* < .001
