# Supplementary figures and images for: Longitudinal relations between parenting stress and child internalizing and externalizing behaviors: Testing within-person changes, bidirectionality and mediating mechanisms
Source: Front Behav Neurosci. 2022 Dec 16;16:942363. doi: 10.3389/fnbeh.2022.942363 (PMC9800797; doi:10.3389/fnbeh.2022.942363)

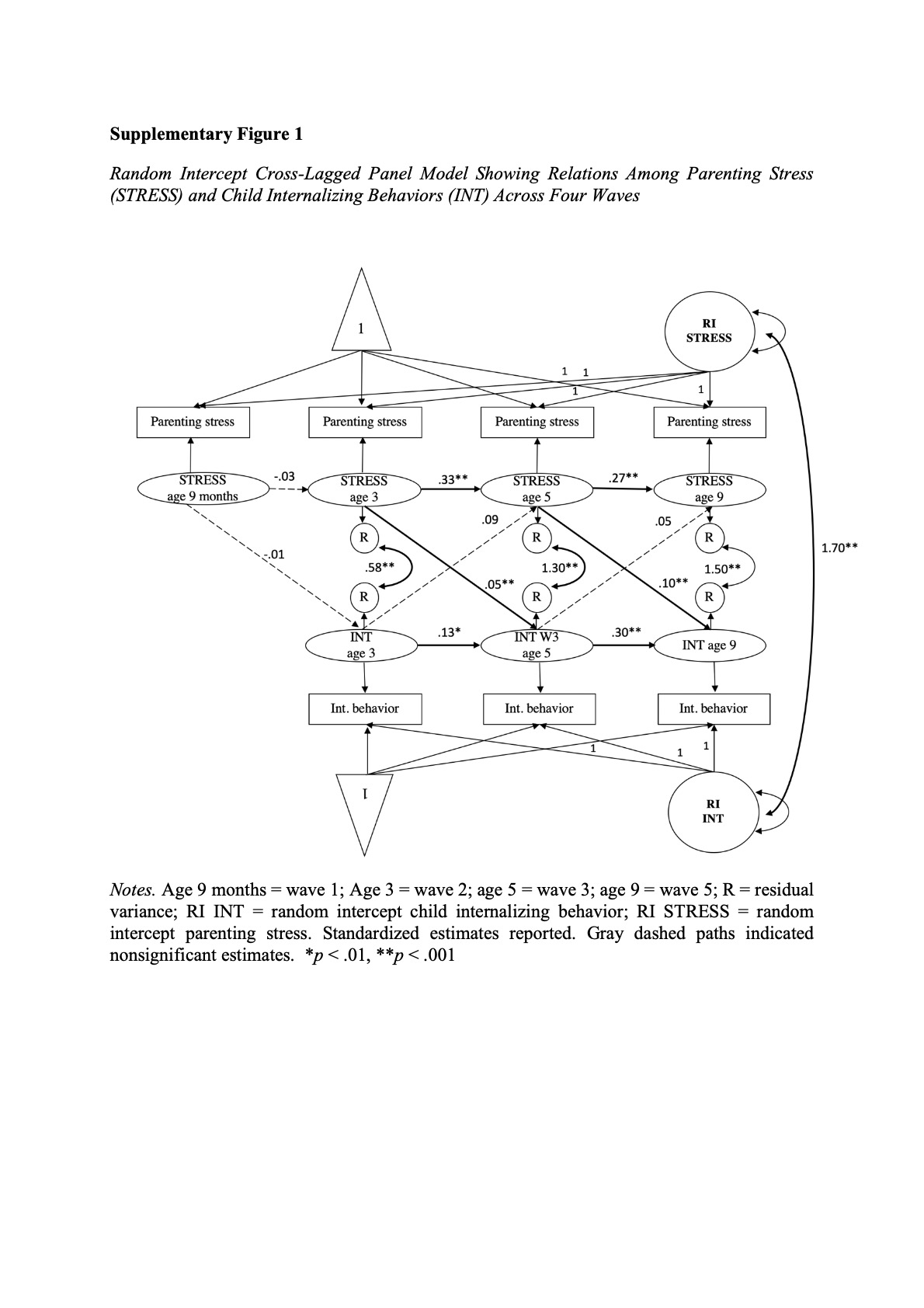

Supplement: Supplementary file 7 [file Image_1.jpg]

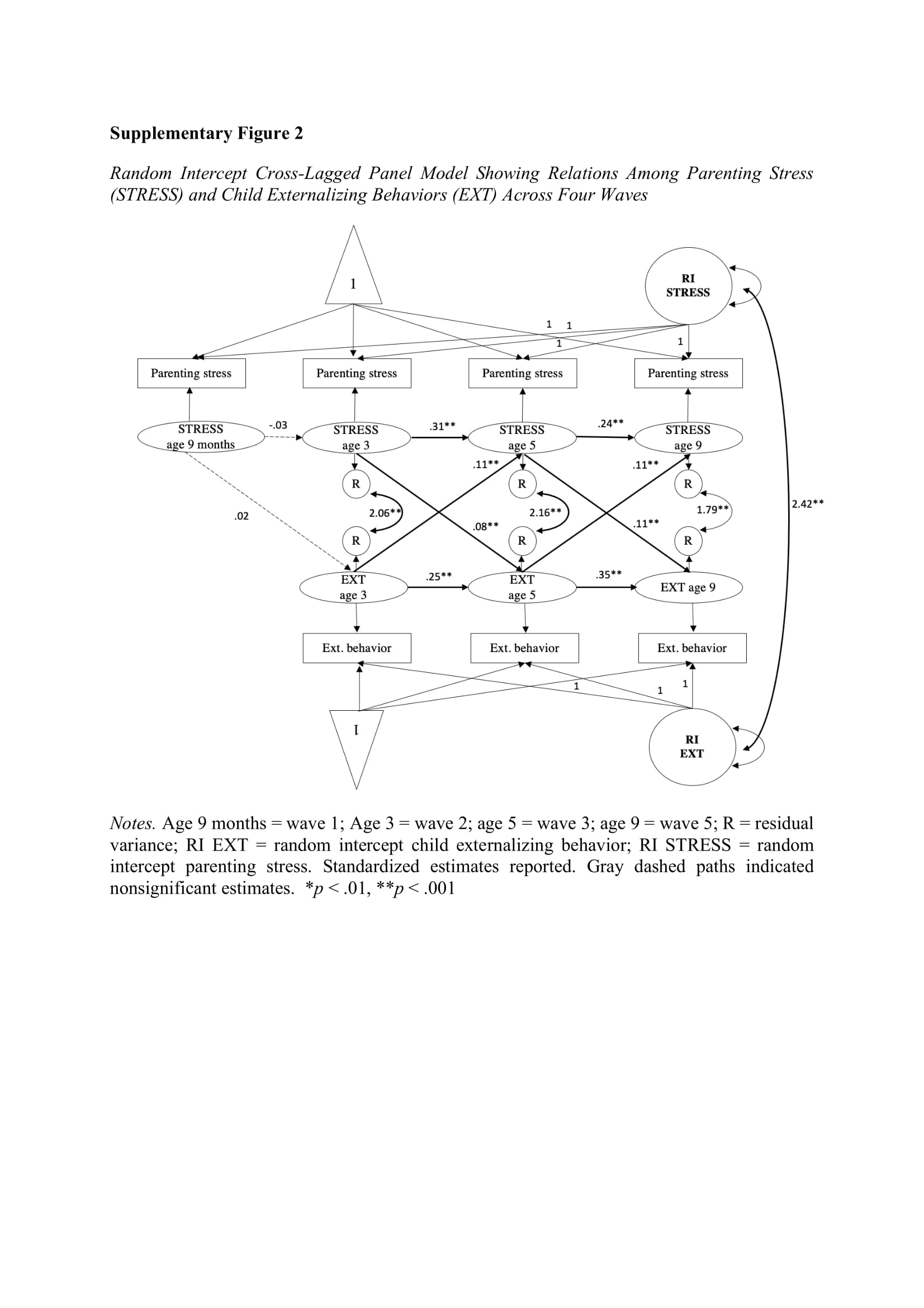

Supplement: Supplementary file 8 [file Image_2.jpg]

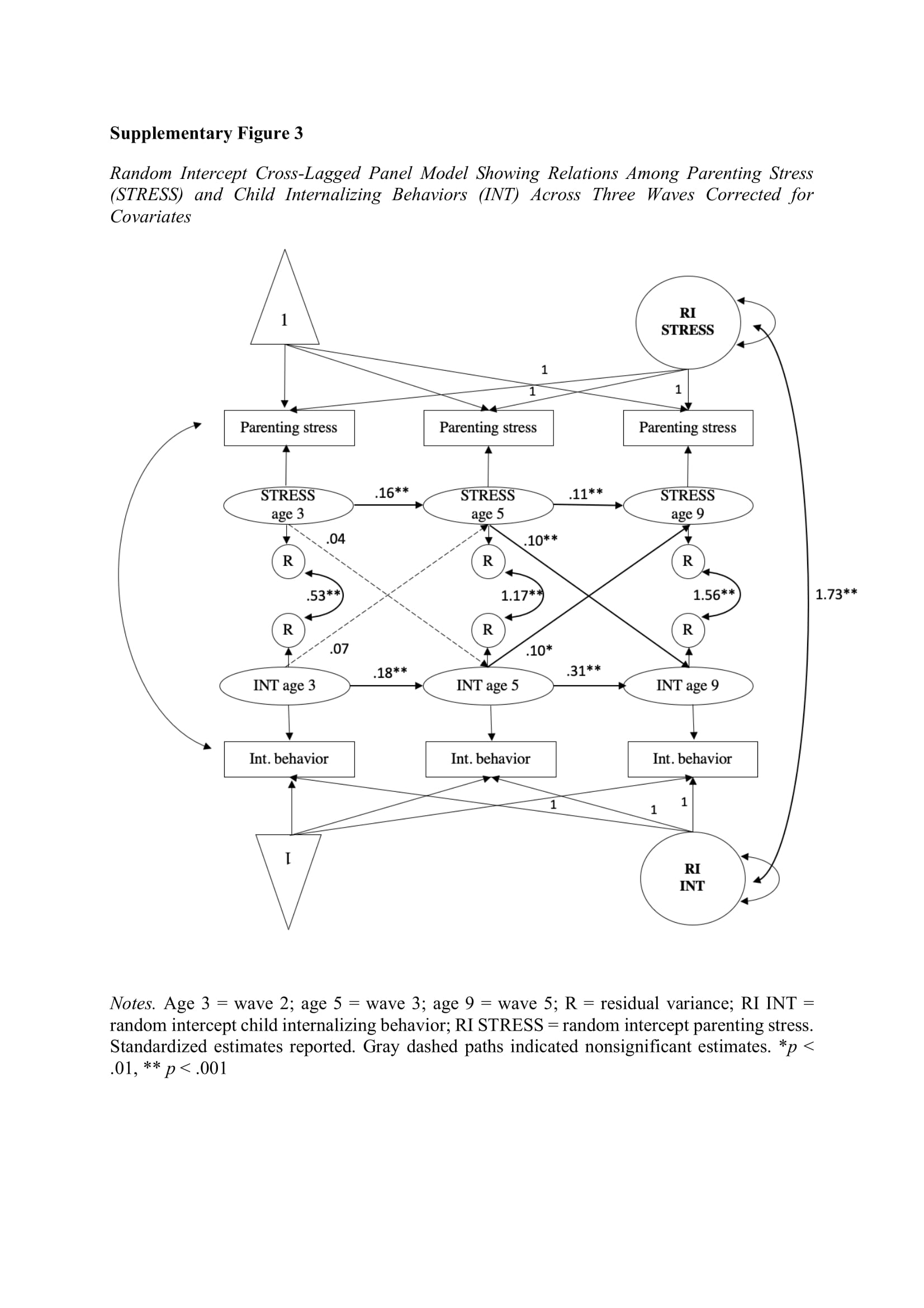

Supplement: Supplementary file 9 [file Image_3.jpg]

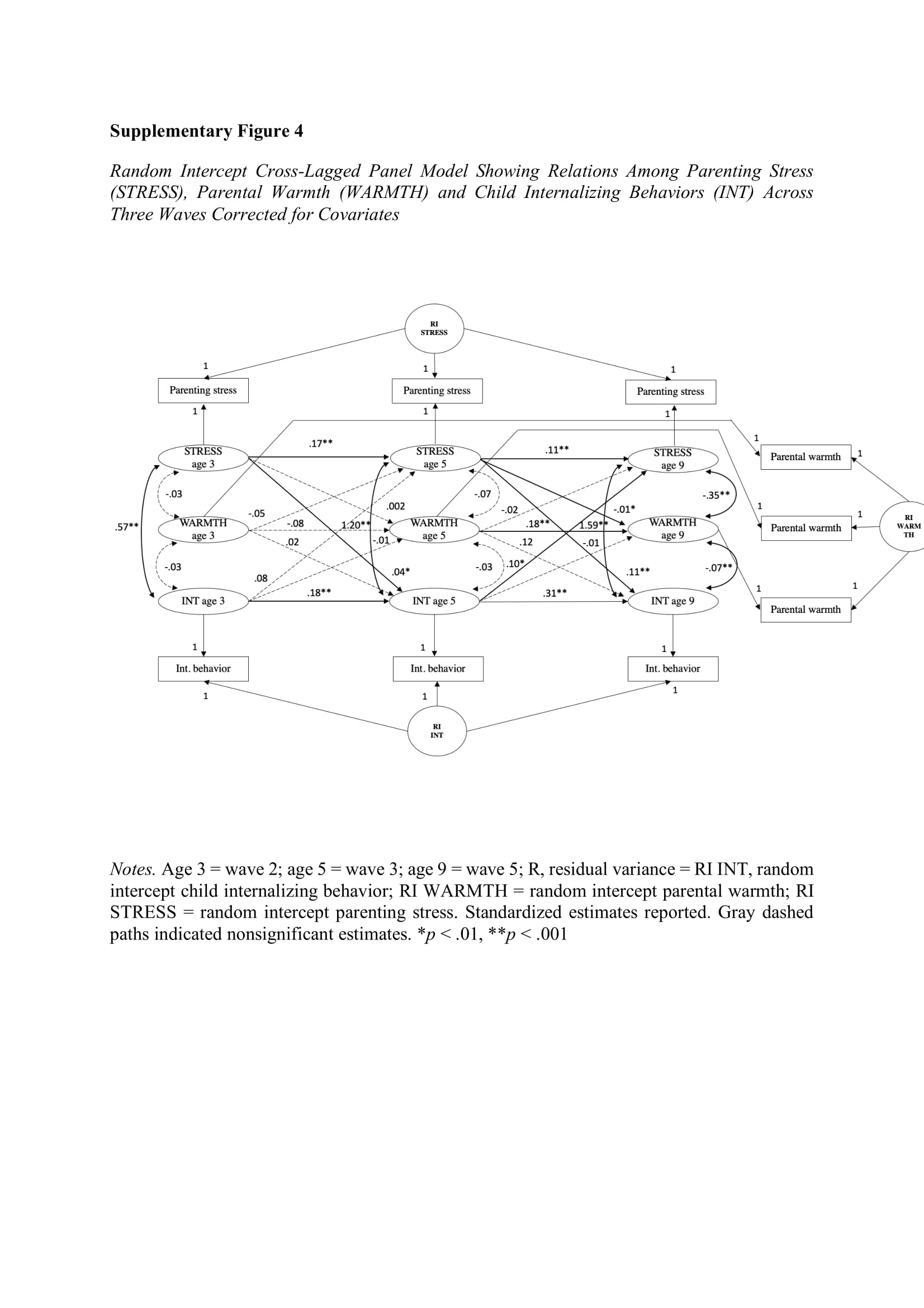

Supplement: Supplementary file 10 [file Image_4.jpg]

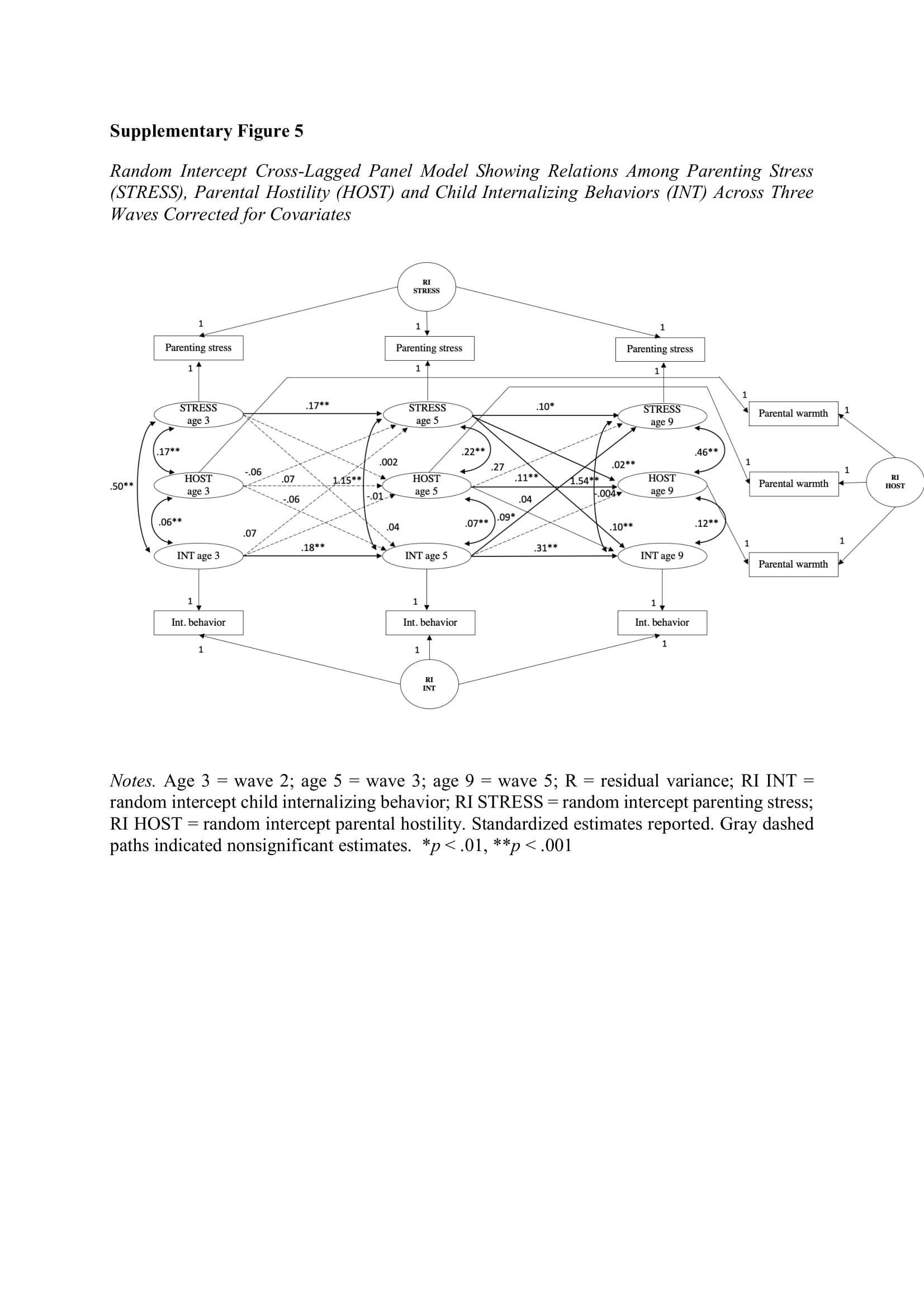

Supplement: Supplementary file 11 [file Image_5.jpg]

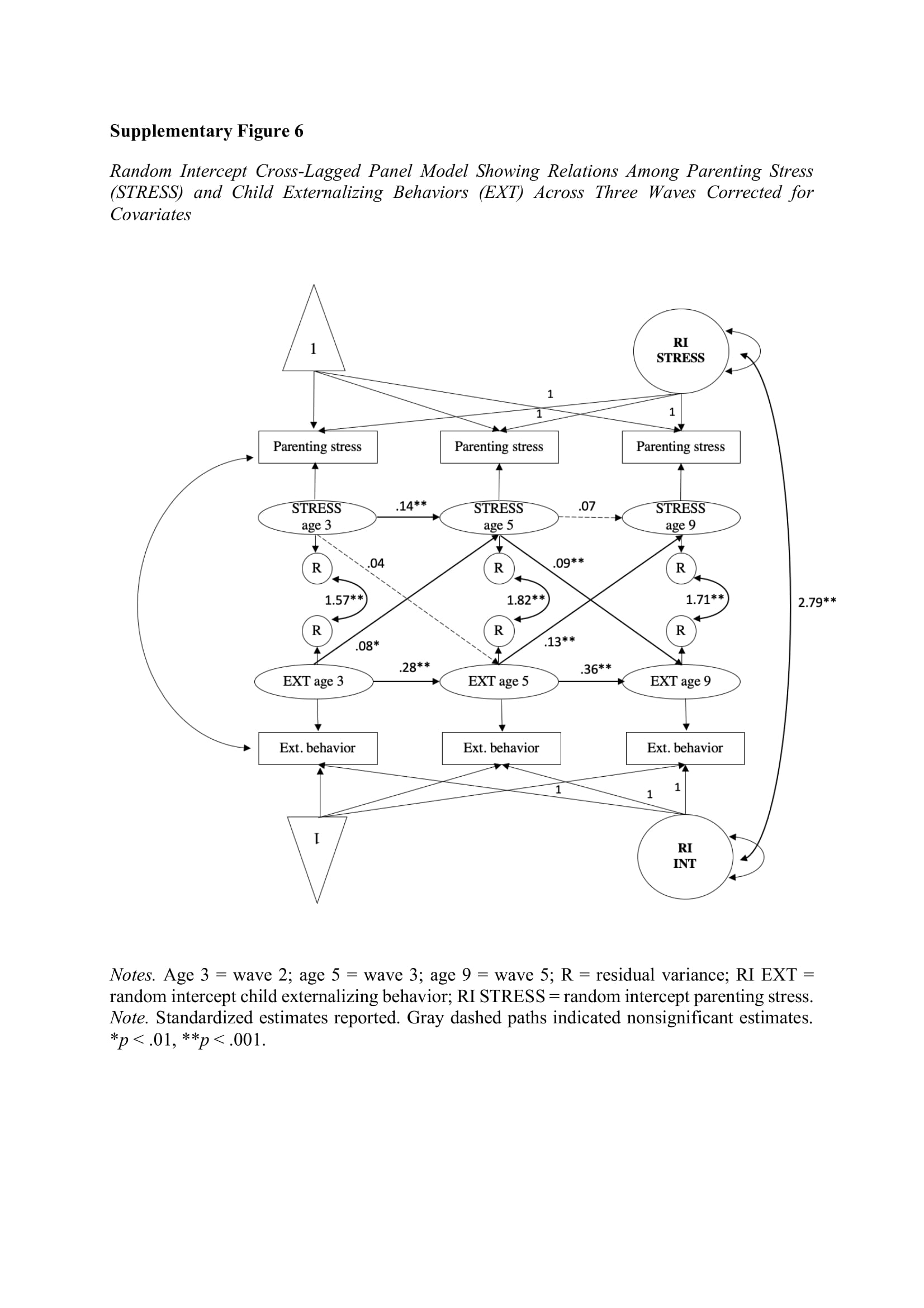

Supplement: Supplementary file 12 [file Image_6.jpg]

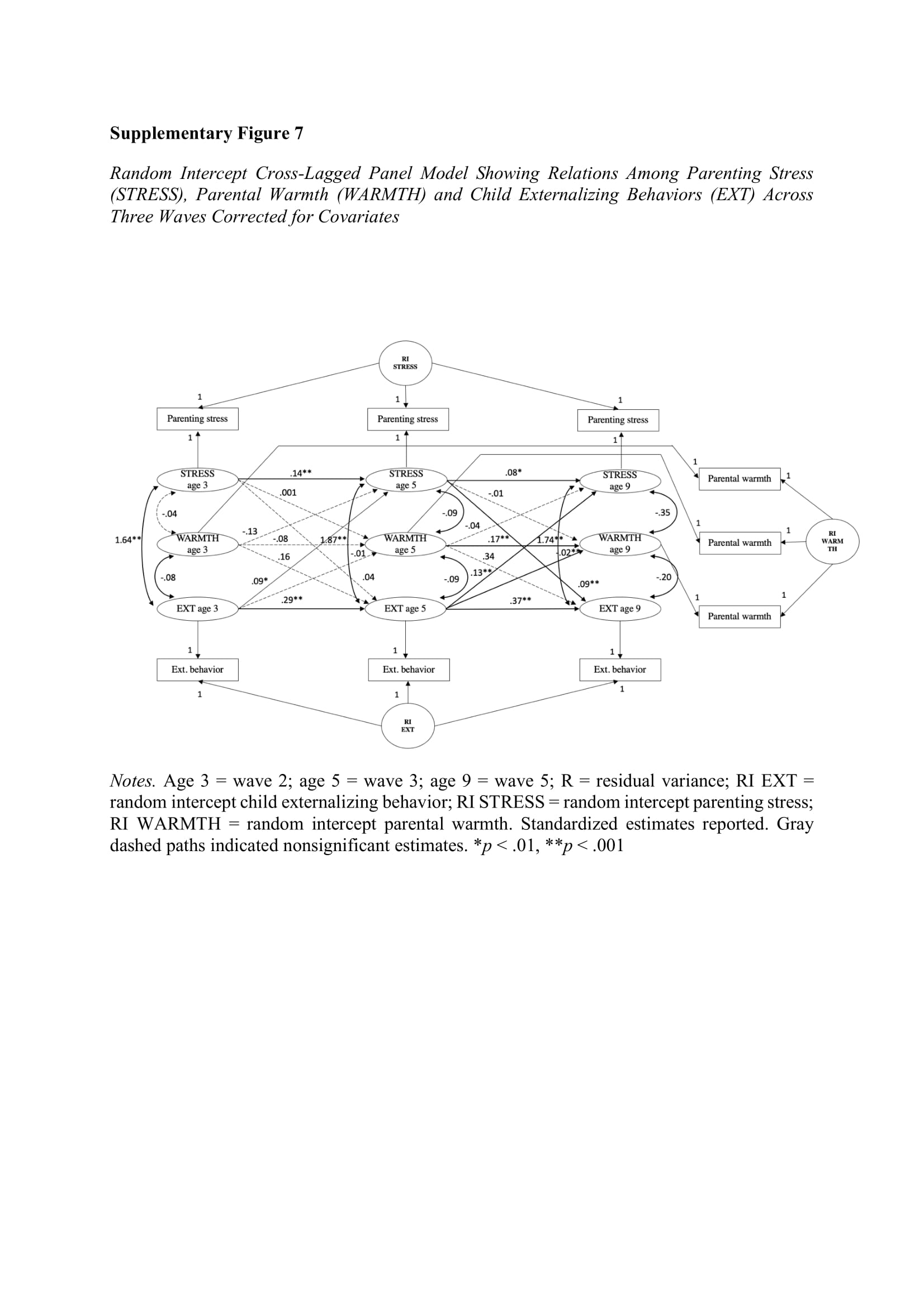

Supplement: Supplementary file 13 [file Image_7.jpg]

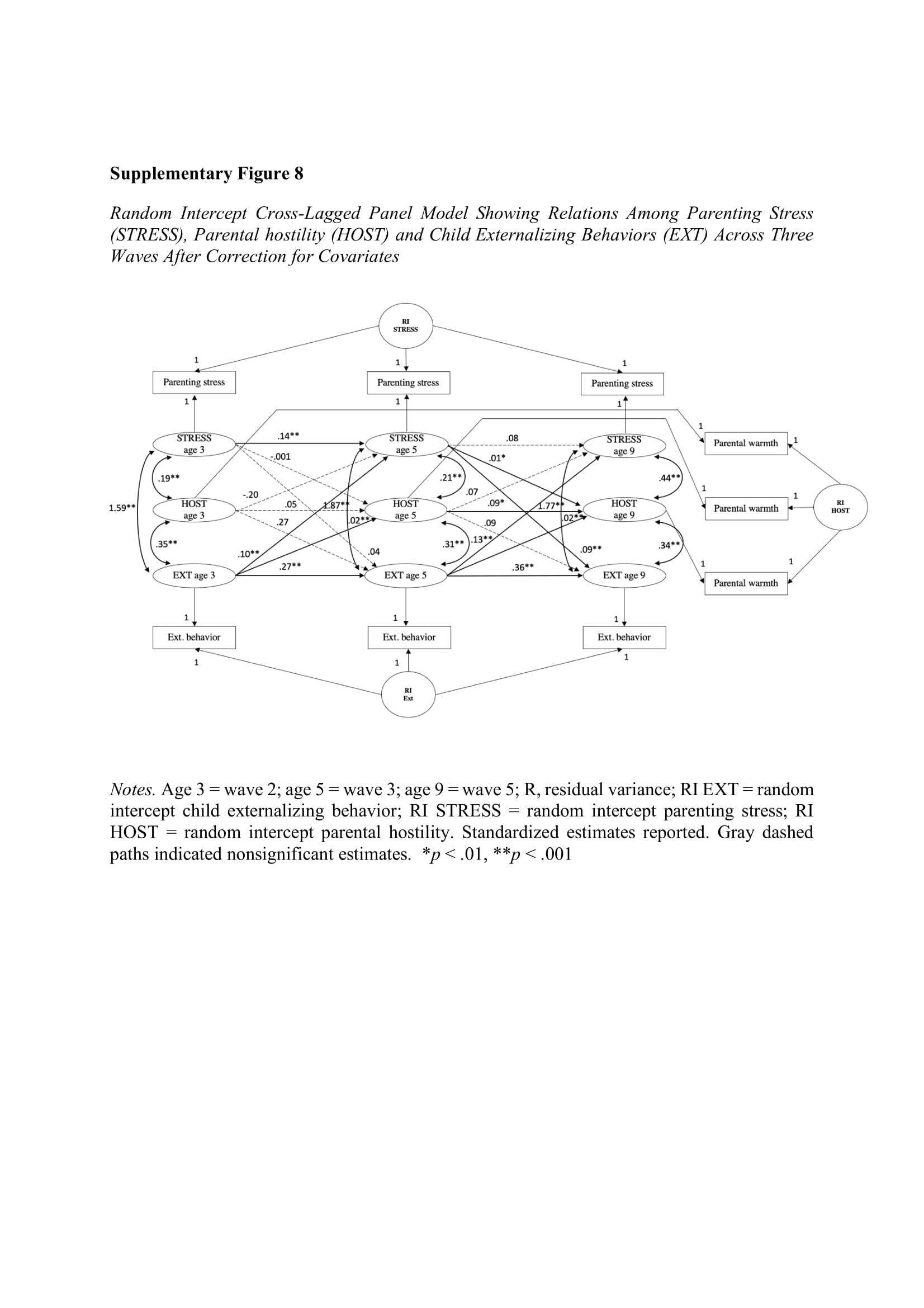

Supplement: Supplementary file 14 [file Image_8.jpg]

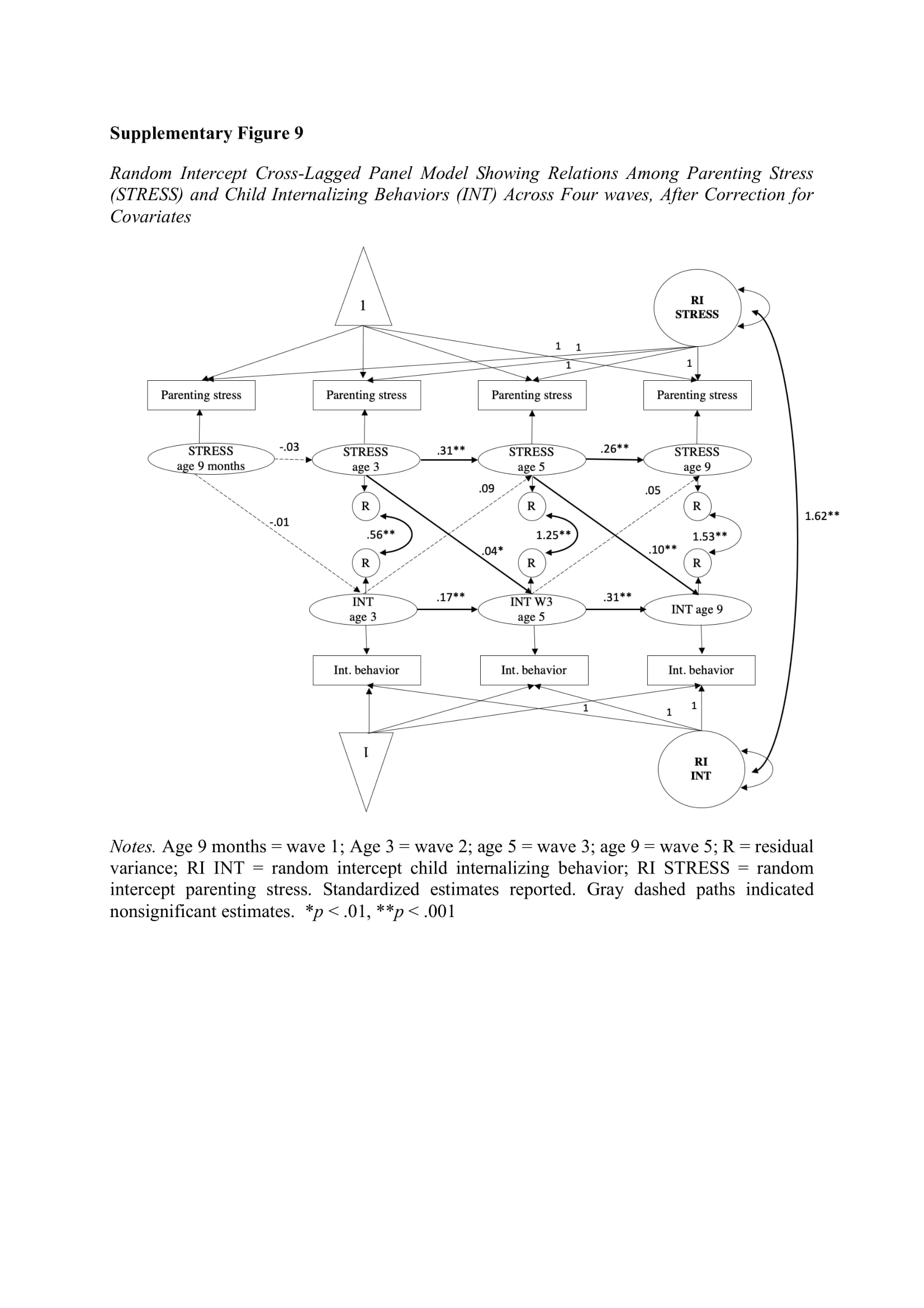

Supplement: Supplementary file 15 [file Image_9.jpg]

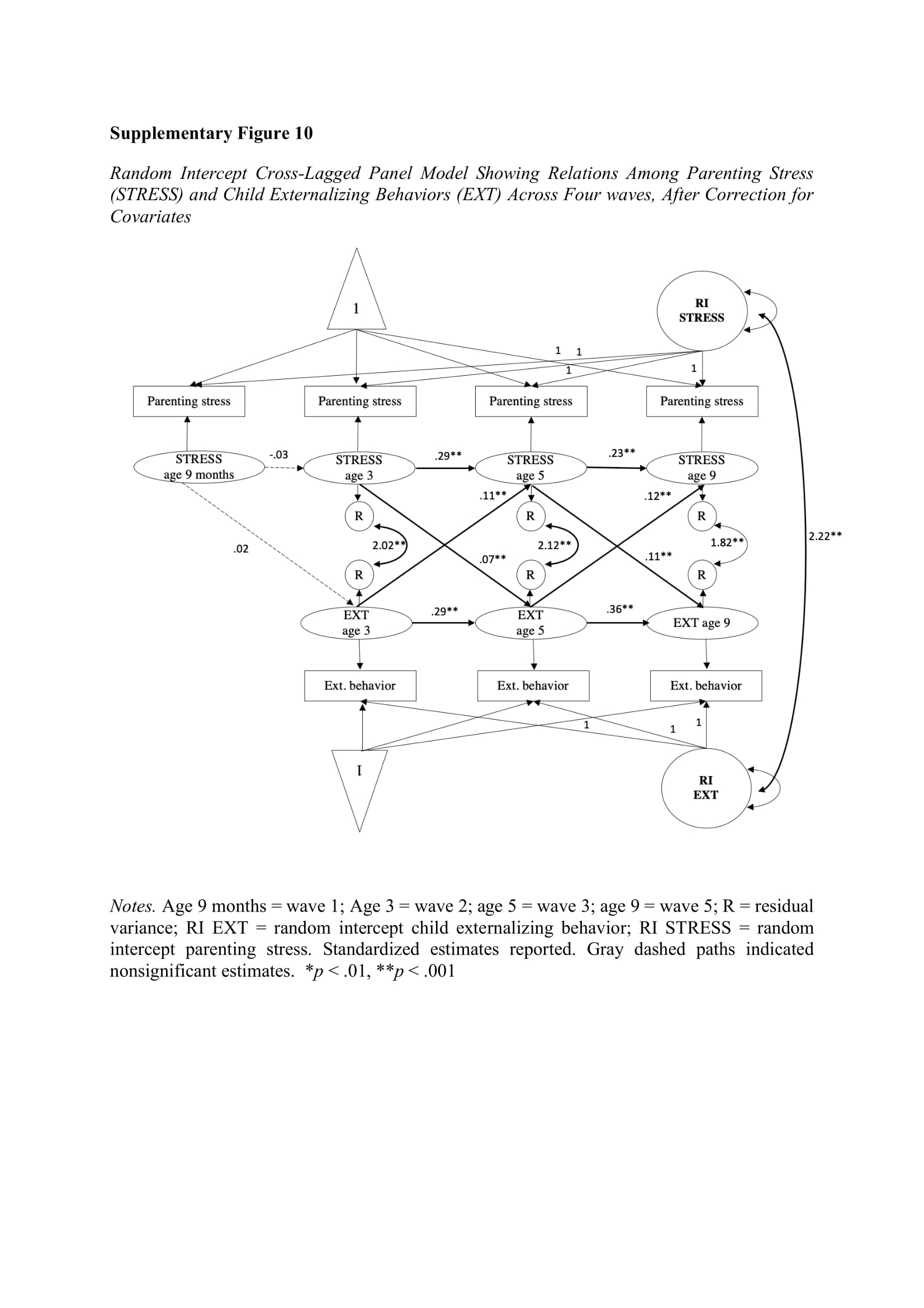

Supplement: Supplementary file 16 [file Image_10.jpg]
